# Radiative Coupled Evaporation Cooling Hydrogel for Above-Ambient Heat Dissipation and Flame Retardancy

**DOI:** 10.1007/s40820-025-01903-0

**Published:** 2025-09-01

**Authors:** Qin Ye, Yimou Huang, Baojian Yao, Zhuo Chen, Changming Shi, Brian W. Sheldon, Meijie Chen

**Affiliations:** 1 School of Energy Science and Engineering, Central South University, Changsha, 430001 People’s Republic of China; 2 School of Engineering, Brown University, Providence, RI 02912 USA

**Keywords:** Radiative cooling, Evaporation cooling, Heat dissipation, Photonic hydrogel, Flame retardancy

## Abstract

By combining the merits of radiative cooling (RC) and evaporation cooling (EC), radiative coupled evaporative cooling (REC) has attracted considerable attention for sub-ambient cooling purposes. However, for outdoor devices, the interior heating power would increase the working temperature and fire risk, which would suppress their above-ambient heat dissipation capabilities and passive water cycle properties. In this work, we introduced a REC design based on an all-in-one photonic hydrogel for above-ambient heat dissipation and flame retardancy. Unlike conventional design RC film for heat dissipation with limited cooling power and fire risk, REC hydrogel can greatly improve the heat dissipation performance in the daytime with a high workload, indicating a 12.0 °C lower temperature than the RC film under the same conditions in the outdoor experiment. In the nighttime with a low workload, RC-assisted adsorption can improve atmospheric water harvesting to ensure EC in the daytime. In addition, our REC hydrogel significantly enhanced flame retardancy by absorbing heat without a corresponding temperature rise, thus mitigating fire risks. Thus, our design shows a promising solution for the thermal management of outdoor devices, delivering outstanding performance in both heat dissipation and flame retardancy.

**Supplementary Information:**

The online version contains supplementary material available at 10.1007/s40820-025-01903-0.

## Highlights

- An all-in-one photonic hydrogel was designed for above-ambient heat dissipation and flame retardancy by sky radiative cooling and evaporation cooling.
- Radiative coupled with evaporation cooling can greatly improve the heat dissipation performance, indicating a 12.0 °C lower than the radiative cooling film under the same conditions.
- Radiative cooling-assisted adsorption can improve atmospheric water harvesting to ensure evaporation under periodic workload and meteorological parameters.

**Supplementary Information:**

The online version contains supplementary material available at 10.1007/s40820-025-01903-0.

## Graphical abstract

**Figure:**
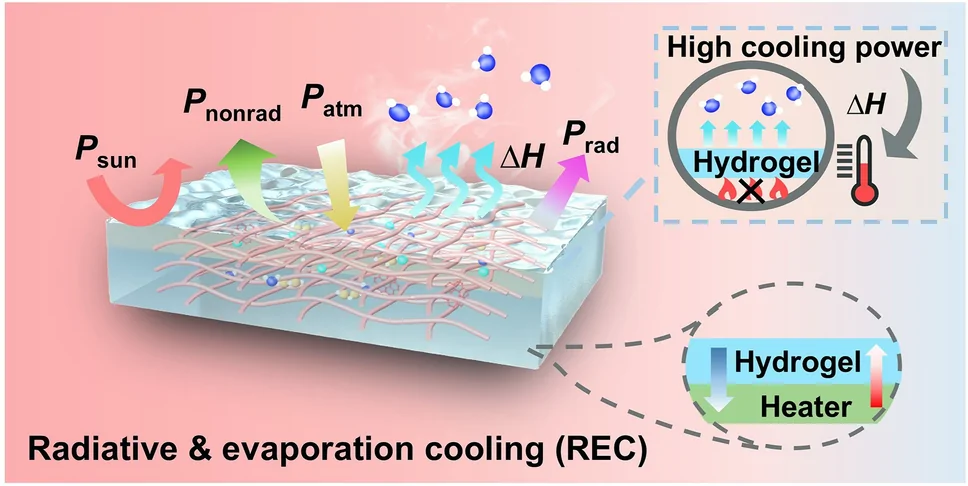

## Introduction

With the increasing power density in electronic devices, efficient heat dissipation has become a critical concern, as operating temperature significantly influences device performance [1, 2], particularly in outdoor equipment exposed to sunlight, such as base stations, transformers, and electronic billboards. Traditional cooling strategies, such as air-conditioners or fans, consume substantial amounts of electricity and cause refrigerant emissions [3, 4]. Additionally, these conventional approaches often result in elevated working temperatures under direct sunlight, especially during peak daylight hours [5, 6]. Consequently, driven by growing awareness of energy efficiency and environmental sustainability, there is an urgent need to develop passive heat dissipation technologies that are both environmentally friendly and energy-efficient [7–9].

Radiative cooling (RC) and evaporation cooling (EC) have been widely explored as energy-saving approaches for heat dissipation [10–12]. RC dissipates heat into outer space (~ 3 K) via the atmospheric long-wave infrared (LWIR) window, requiring materials with high thermal emittance (${\overline{\varepsilon }}_{\mathrm{LWIR}}$) and high solar reflectance (${\overline{R} }_{\mathrm{solar}}$) [13–15]. Materials such as SiO_2_ [16, 17], hBN [18], PVDF-HFP [19, 20], and PDMS [21] possess minimal solar absorption but high intrinsic mid-infrared absorption, which, when combined with scattering structures [22, 23] (e.g., porous [17, 24], sphere [25, 26], hollow [27, 28], fiber [29, 30], core–shell [31]), enhance cooling performance. However, RC faces limitations, including thermal stability concerns at elevated temperatures due to polymer degradation [32, 33], and restricted cooling power (typically below 150 W m⁻^2^ at room temperature), which is further influenced by environmental factors like humidity, cloudiness, and rainfall [34]. EC utilizes the latent heat of water evaporation, providing high cooling power [35–37]. However, in outdoor daytime, water has an unavoidable solar absorption, which makes it difficult to cool down the outdoor device under sunlight. In addition, the passive water supply also limits its practical application since the additional active water replenishment structures will increase the cost and structural complexity [38].

Combining RC and EC, known as radiative evaporative cooling (REC), significantly enhances daytime cooling performance by simultaneously exploiting high solar reflectance, thermal emittance, and latent heat release [39–41]. Recent studies have focused primarily on sub-ambient REC applications [42, 43], which could achieve ~ 3.3 °C lower than pure RC [44]. While little attention was paid to the above-ambient heat dissipation application, the passive water supply at such a high working temperature is also a challenge. It is urgent to develop REC technology in the above-ambient heat dissipation to improve its stability under high working temperatures.

Hence, REC was developed in this work for above-ambient heat dissipation and flame retardancy by multi-mode cooling strategies, which was achieved by an all-in-one photonic hydrogel consisting of PDMAPS, LiCl, hBN nanoplates, and Al_2_O_3_. In the daytime with a high workload, RC coupled with EC can greatly improve the heat dissipation performance; while in the nighttime with a low workload, RC-assisted adsorption can improve atmospheric water harvesting (AWH) to ensure EC in the daytime. In addition, the latent heat storage capability of the hydrogel enhances flame retardancy, mitigating fire risks by absorbing heat without a significant temperature increase. This approach provides a promising strategy for environmentally friendly and energy-efficient passive heat dissipation.

## Experimental Section

### Materials

[2-(Metharyloyloxy)ethyl]dimethyl-(3-sulfopropyl)ammonium hydroxide (DMAPS), polyethylene glycol diacrylate (PEGDA, average Mn = 575), lithium chloride (LiCl), and ammonium persulfate (APS) were purchased from Shanghai Macklin Biochemical Co., Ltd. hBN nanoplates with the average particle size of 500 nm and aluminum (Al_2_O_3_) with average particle size of 500 nm were purchased from Yaotian Nano Co., Ltd.

### Fabrication of REC Hydrogel

Typically, based on the previous work [45], 7 g of DMAPS powder was mixed with 3 g of deionized water, 0.64 g of hBN, and 0.22 g of aluminum. After dissolving completely in the solution, 0.06 g of APS as an initiator and 70 µL of PEGDA as a crosslinker were added to the mixture. Then, the mixture was sonicated for 5 min and polymerized at ~ 60 °C for ~ 12 h. The hydrogel was immersed in deionized water for 12 h to remove unreacted substances, and the pure poly-[2-(Metharyloyloxy) ethyl]dimethyl-(3-sulfopropyl)ammonium hydroxide (PDMAPS) hydrogel was obtained. To embed sorbent LiCl into the pure PDMAPS hydrogel, the PDMAPS hydrogel was soaked in 4 M LiCl solution for ~ 24 h. The water content ratio can be obtained by the ratio of the current water content to the saturated water content in the hydrogel.

### Characterizations

Scanning electron microscope (SEM) images were conducted by a MIRA4 LMH TESCAN scanning electron microscope (5 kV). Sample thickness was measured by the micrometer. A differential scanning calorimeter (DSC, NETZSCH-241) was used to measure the evaporation enthalpy. The mass change at different temperatures was measured using a thermogravimetric analyzer (TGA, Rigaku/TG-DTA8122). Thermal conductivity was measured using a Hot Disk (TPS2500S). X-ray diffraction result (XRD) was measured using the Cu target at 45 kV and 40 mA (PANalytical, Empyren), and Fourier-transform infrared (FTIR) spectroscopy was measured by a Nicolet iS50 FTIR spectrometer. The tensile test was conducted by the Jitai Series Universal Tensile Testing Machine.

Spectral reflectance was measured by spectrometers, which were divided into the short wavelengths (0.3–1.7 μm) and the long wavelengths (1.7–17 μm). In the first short wavelength region, the micro-spectrometer from ideaoptics (PG2000 for 0.3–1.0 μm, NIR1700 for 1.0–1.7 μm) was used with an integrating sphere (IS-50–10-R), and a diffuse standard whiteboard (JY-WS1) was used as a reference. In the second long wavelength region, a Fourier-transform infrared spectrometer (Nicolet iS50) was used with an MCT detector and a gold integrating sphere (Pike Technologies), and a diffusive gold sample was used as a reference.

### Field Tests

Indoor water capture and release experiments were conducted in the laboratory, and the ambient temperature (*T*_amb_) and relative humidity (RH) were controlled using a constant temperature and humidity chamber (HWS-80B). A heater was used to simulate the workload, which was controlled by a DC power supply. An electronic balance from Sartorius was used to measure the mass change of the tested sample at each period. After each experiment, the sample’s water content was initialized to its initial value by heating for the next experiment. Flame retardancy was conducted by an alcohol lamp, and the tested sample was exposed to flame. Interior temperatures were measured by T-type thermocouples, and optical, infrared images were recorded by optical and infrared cameras (Fluke Ti400).

In outdoor experiments, a shield with aluminum foil was used to eliminate radiative cooling and allow airflow for ambient temperature measurement. All samples were placed on the foam. Temperatures of samples at the bottom were measured using T-type thermocouples, and RH was monitored by a humidity recorder (Suzhou Tasi Electronics Co., Ltd.). Solar intensity was measured using a solar meter (TES-1333r, TES), and all temperature data were recorded by a laptop using a data acquisition device (34970A, Keysight).

## Results and Discussion

### Working Principle of REC Hydrogel for Heat Dissipation and Flame Retardancy

**Fig. 1:**
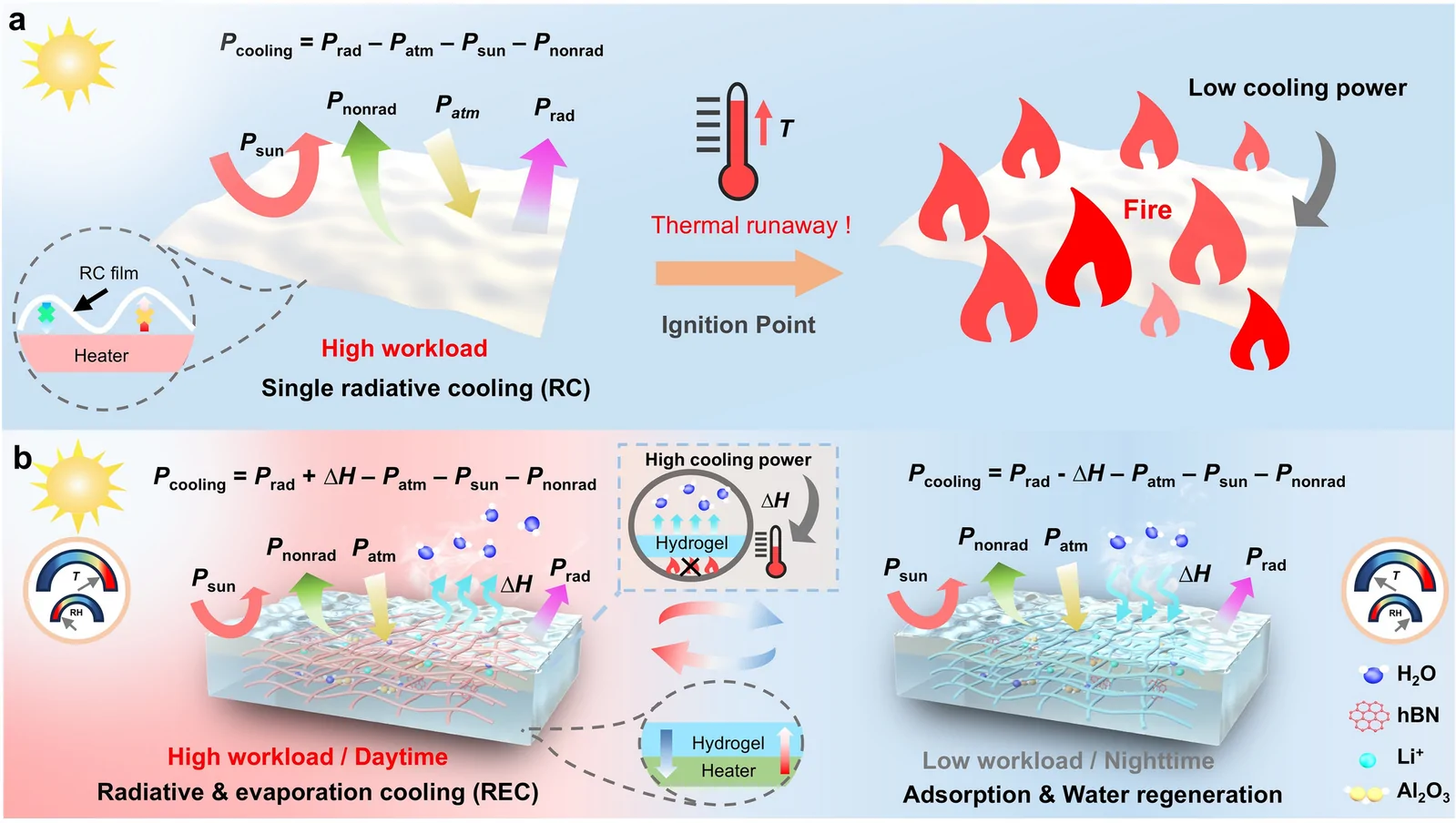
Working principle of REC hydrogel. **a** Traditional polymer RC film for above-ambient heat dissipation, which easily ignites at the ignition point, resulting in weakened cooling performance. **b** All-in-one REC hydrogel for above-ambient dissipation. In the daytime with a high workload, REC maximizes passive heat dissipation performance under the high ambient temperature (*T*_amb_) and low relative humidity (RH). In the nighttime with a low workload, water can be harvested from the atmosphere by RC-assisted adsorption for the water cycle under the low *T*_amb_ and high RH

Traditional RC film typically consists of polymers, such as PMMA, PDMS, PVDF, and so on [46]. However, these materials can limit above-ambient heat dissipation due to increased thermal contact resistance caused by voids between the RC film and the substrate (Fig. 1a). In addition, these polymer-based RC films face operational temperature constraints because of their relatively low softening and ignition temperatures, potentially compromising their performance and increasing fire risk. To address these limitations, a REC hydrogel was designed here consisting of hydrogel PDMAPS, hygroscopic salt (LiCl), and hBN nanoplates, as well as Al_2_O_3_ (Fig. 1b). The dielectric particles hBN serve as efficient scatters, significantly enhancing solar reflectance (${\overline{R} }_{\mathrm{solar}}$). LiCl balances moisture adsorption and desorption processes under periodic workload and meteorological parameters, for example: during the daytime under high workload conditions, RC coupled with EC substantially improves heat dissipation performance, while at night with a low workload, RC-assisted adsorption enhances atmospheric water harvesting (AWH) to replenish water for daytime evaporative cooling. In addition, the latent heat stored within the REC hydrogel considerably improves flame retardancy by absorbing and releasing heat without raising the material temperature, thus significantly reducing the fire risk.

Based on the designed working principle of REC hydrogel, a theoretical analysis based on the energy balance law was conducted initially to evaluate its above-ambient heat dissipation and water cycling performance. The detailed calculation can be found in Supporting Information S1.

**Fig. 2:**
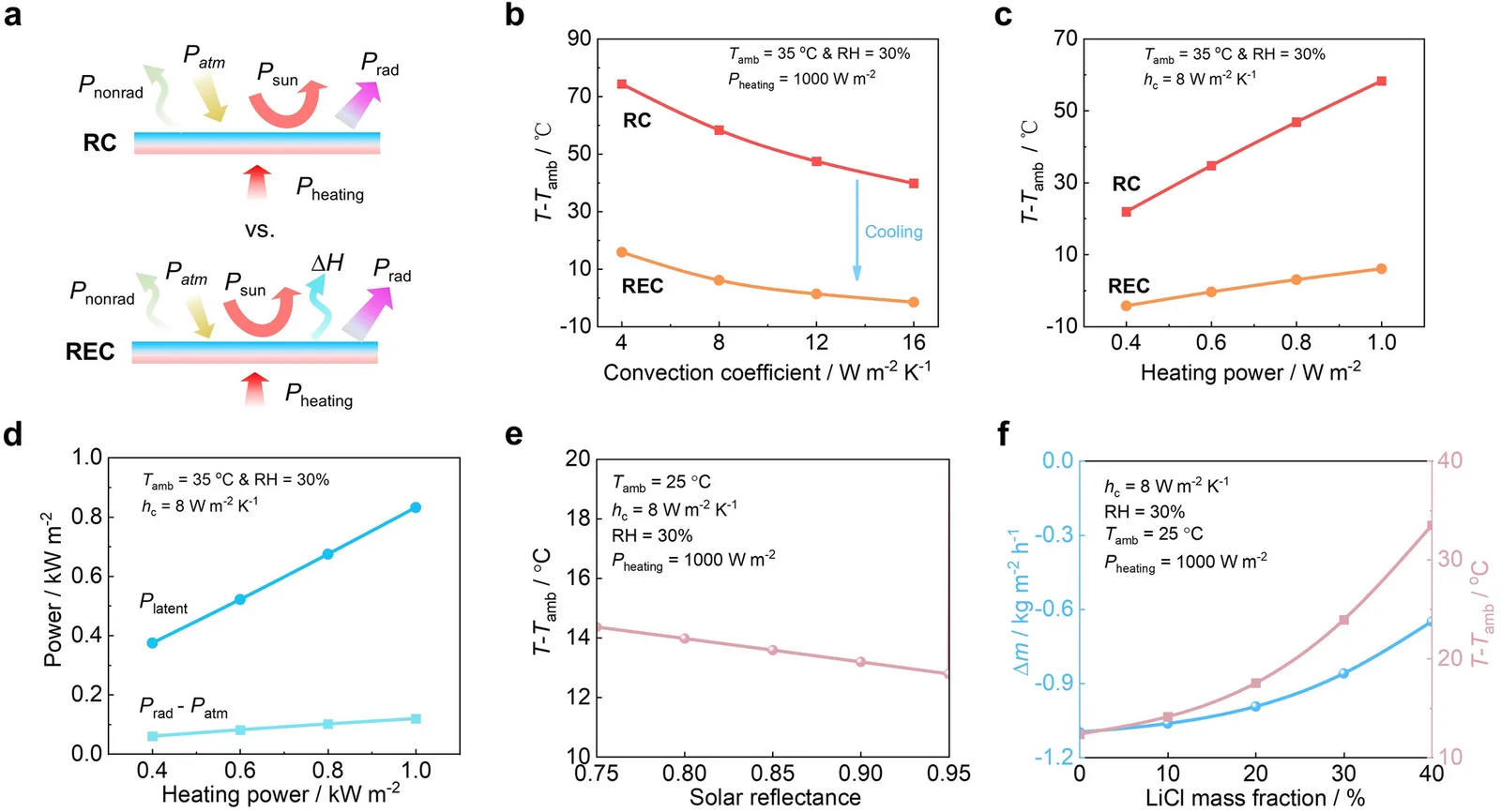
Theoretical analysis of heat dissipation performance based on REC. **a** Schematic diagram of heat flow in RC and REC. Temperature change for the ideal RC and REC at **b** different convection coefficients *h*_c_ (ambient temperature *T*_amb_ = 35 °C, relative humidity RH = 30%, and heating power *P*_heating_ = 1000 W m^−2^) and **c** different *P*_heating_ (*T*_amb_ = 35 °C, RH = 30%, and *h*_c_ = 8 W m^−2^ K). **d** Detailed power for latent heat (*P*_latent_), and RC power (*P*_rad_—*P*_atm_) at different *P*_heating_ in the ideal REC (*T*_amb_ = 35 °C, RH = 30%, and *h*_c_ = 8 W m^−2^ K^−1^). **e** Temperature change of the REC under different solar reflectance (*T*_amb_ = 25 °C, RH = 30%, *h*_c_ = 8 W m^−2^ K^−1^, and *P*_heating_ = 1000 W m^−2^). **f** Mass and temperature changes of the REC under different LiCl mass fraction (*T*_amb_ = 25 °C, RH = 30%, *h*_c_ = 8 W m^−2^ K^−1^, and *P*_heating_ = 1000 W m^−2^). Here, the ideal RC and REC had near-perfect solar reflectance (reflectance = 1 in wavelength λ = 0.3–2.5 µm) and mid-infrared thermal emittance (thermal emittance = 1 in wavelength λ > 2.5 µm), and the liquid–gas transition latent heat was equal to that of pure water

The working principle of RC and REC is shown in Fig. 2a**.** Ideal spectra of RC and REC were assumed as ${\overline{R} }_{\mathrm{solar}}$ = 1 and ${\overline{\varepsilon }}_{\mathrm{LWIR}}$ = 1 with a broadband thermal emittance. Compared with pure RC, the REC can greatly improve heat dissipation due to the latent heat release at different thermal convection coefficients (*h*_c_) (Fig. 2b). For example, the temperature change of REC could be 52.2 °C lower than that of RC at the heating power (*P*_heating_) of 1000 W m^−2^ (58.3 vs. 6.1 °C). Furthermore, when *P*_heating_ increased from 400 to 1000 W m^−2^, the temperature change of RC increased rapidly from 21.9 to 58.3 °C while the REC only increased from −4.3 to 6.1 °C (negative: sub-ambient; positive: above-ambient) in Fig. 2c. It can be found that the large *P*_heating_ could greatly improve the power of latent heat (*P*_latent_) compared with the RC power (*P*_rad_–*P*_atm_) due to the large enthalpy and evaporation rate of water (Fig. 2d). The temperature change of REC only increased slightly from 12.8 to 14.4 °C when ${\overline{R} }_{\mathrm{solar}}$ deceased from 0.95 to 0.75 due to unavoidable intrinsic solar absorption of water (Fig. 2e). These results indicate that the REC hydrogel maintains excellent heat dissipation performance across different water content levels.

To ensure a practical-level application, we take meteorological parameters such as wind speed (i.e., convection), RH, and *T*_amb_ into consideration. This is because these parameters greatly impact passive heating dissipation. In terms of the convective effect, increasing *h*_c_ was conducive to EC as well as thermal convection at the above-ambient heat dissipation. When *h*_c_ increased from 4 to 16 W m^−2^ K^−1^, the temperature change of REC decreased from 15.8 to −1.6 °C (Fig. 2b), resulting in a great heat dissipation performance due to the coupled effect of EC and thermal convection.

High RH impaired RC performance due to the reduced atmospheric transmittance window (Fig. S1a). Hence, higher RH was not conducive to above-ambient heat dissipation, which increased the temperature change of REC from 6.1 (RH = 30%) to 10.7 °C (RH = 70%) (Fig. S1b). In addition, high RH increased the saturated vapor pressure in the air, while the elevated working temperature of the REC hydrogel raised the saturated vapor pressure within the sample. The net effect of these two factors resulted in a relatively consistent difference in saturated vapor pressure between the sample and the environment, thereby maintaining stable mass change and EC performance (Fig. S1c). Therefore, the reduced atmospheric transmittance at high RH played a critical role in suppressing heat dissipation performance. However, since most devices operate during the daytime when RH is relatively low, the REC-based system benefits from improved heat dissipation under these conditions.

Increasing *T*_amb_ attenuates atmospheric transmittance (Fig. S2a) and raises the saturated vapor pressure in the air. This results in a high working temperature of REC (Fig. S2b). For example, the working temperature of REC increased from 34.8 °C (*T*_amb_ = 15 °C) to 42.4 °C(*T*_amb_ = 35 °C) while its temperature change decreased from 19.8 °C (*T*_amb_ = 15 °C) to 7.4 °C (*T*_amb_ = 35 °C), which could be attributed to the enhanced mass change or EC performance at the high *T*_amb_ due to an increased saturated vapor pressure difference (Fig. S2c). REC shows a better passive heat dissipation effect over a wide range of meteorological parameters (*h*_c_, RH, and *T*_amb_).

Beyond heat dissipation performance, water capture performance in the nighttime with a low workload is also critical for the water cycle in REC. Such performance could be enhanced by adsorbent LiCl, which could reduce the vapor pressure by tuning the LiCl mass fraction (Fig. S3a) and improve the water capture performance in the nighttime with a low workload (Fig. S3b). LiCl would weaken EC in the daytime with a high workload, while it maintained a relatively long-term EC performance at the same water content, for example, the calculated evaporation rate dropped from 1.10 to 0.65 kg m^−2^ h^−1^ when LiCl mass fraction increased from 0 to 40 wt% (Fig. 2f). Increasing *T*_amb_ and RH promoted the water capture performance due to the raised saturated vapor pressure difference between the sample and the environment (Fig. S3c, d). In addition, periodic workload or adsorbent concentration can be determined to maintain the passive water cycle based on meteorological parameters, for example, at a constant heating power of 300 W m^−2^, a LiCl mass fraction of 40% could balance the water capture and release (Fig. S4). Therefore, based on the periodic changes in meteorological parameters and workload, the heat dissipation of outdoor devices could be greatly enhanced by passive REC.

### Fabrication and Characterization of REC Hydrogel

**Fig. 3:**
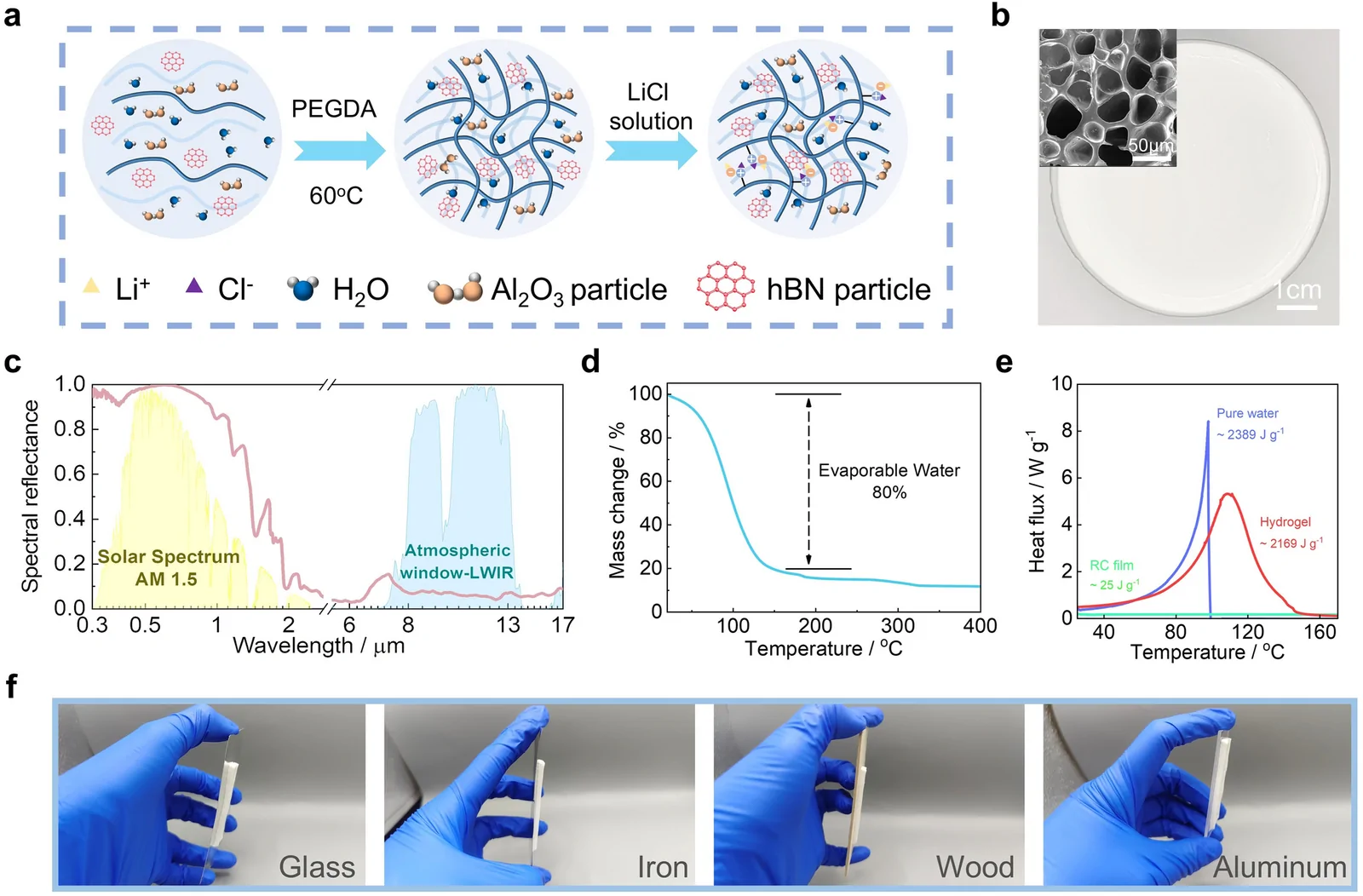
Characterization of all-in-one REC hydrogel. **a** Schematic diagram of the all-in-one hydrogel for REC, including polymer chain, scatter hBN, Al_2_O_3_ particles, and adsorbent LiCl. **b** Optical and scanning electron microscopy (SEM) images at the top surface of REC hydrogel. **c** Spectral reflectance of REC hydrogel with a water content of 5 wt%. Backgrounds are the normalized solar irradiance (yellow) and atmospheric transmittance windows (cyan). **d** Thermogravimetric analysis (TGA) curve of REC hydrogel. **e** Differential scanning calorimetry (DSC) curves of pure water and water in REC hydrogel to evaluate latent heat. **f** Optical images of REC hydrogel attached to different substrates, including glass, iron, wood, and aluminum

A dual-ion hydrogel PDMAPS was used as the AWH medium [45], serving as a matrix for incorporating hBN, Al_2_O_3,_ and LiCl salt to prepare the REC hydrogel (Fig. 3a). Detailed preparation information can be found in the above experiment section. The LiCl was attached by soaking the hydrogel in the LiCl solution (4 M) for over 24 h for saturated adsorption, which could maintain the high-water storage compared with the hydrogel without LiCl (Fig. S5). The concentration of LiCl solution had little effect on the optical properties at the same water content, while the water capture performance could be enhanced at high concentrations of LiCl solution (Fig. S6), which can be determined to balance the water capture and release at different heating powers as discussed in Fig. S4.

After incorporating hBN, Al_2_O_3_, and LiCl, the REC hydrogel exhibited a uniform white color and element distribution, and scanning electron microscope (SEM) images from the top and cross-section surfaces (Figs. 3b and S7) in this porous structure. This porosity increases the contact area between the hydrogel and the surrounding air, enhancing water storage, capture, and release, thereby optimizing EC performance. In addition, the X-ray diffraction results (XRD) exhibit that the sample shows characteristic peaks of hBN and Al_2_O_3_ without any characteristic peaks of LiCl crystal, indicating the existence of the coordinate salt (Fig. S8a). The peaks at ~ 1033 cm^−1^ and ~ 1164 cm^−1^ represent the symmetric and asymmetric stretching vibrations of S = O, and the –C = C– (~ 962 cm^−1^), –CH_2_– (~ 1472 cm^−1^), –CH_3_ (~ 1375 cm^−1^) groups were observed on Fourier-transform infrared (FTIR) spectra (Fig. S8b), indicating that strong interaction exists within the hydrogel.

In this designed hydrogel, ${\overline{R} }_{\mathrm{solar}}$ could be largely enhanced from 0.074 to 0.808 at a water content of 20 wt% when the mass fraction of scatter hBN (*m*_hBN_) in the hydrogel increases from 0 to 6 wt%, while ${\overline{\varepsilon }}_{\mathrm{LWIR}}$ changed little due to the intrinsic absorption of polymer and water (Fig. S9). Further hBN addition could slightly increase ${\overline{R} }_{\mathrm{solar}}$ = 0.847 at *m*_hBN_ = 9 wt%, and a higher solid content will weaken its moisture capture performance. The introduction of Al_2_O_3_ particles served as a plasticizer to improve the mechanical properties of the photonic hydrogel [43], with better stretchability compared with the hydrogel without Al_2_O_3_ particles (Fig. S10). The addition of Al_2_O_3_ had a slight effect on ${\overline{R} }_{\mathrm{solar}}$, while it would lead to a decrease in water capture performance due to the increased non-hygroscopic Al_2_O_3_ particles (Fig. S11). When the thickness of the hydrogel increased from 3 to 9 mm at a water content of 20%, ${\overline{R} }_{\mathrm{solar}}$ only increased slightly from 0.806 to 0.839 and ${\overline{\varepsilon }}_{\mathrm{LWIR}}$ did not change obviously due to the saturated absorption of water and polymer (Fig. S12). The amount of water content in the hydrogel would also affect ${\overline{R} }_{\mathrm{solar}}$. When water content increased from 10wt% to 80 wt%, ${\overline{R} }_{\mathrm{solar}}$ decreased from 0.851 to 0.627 due to the intrinsic absorption of water and ${\overline{\varepsilon }}_{\mathrm{LWIR}}$ did not change obviously (Fig. S13). As discussed above, ${\overline{R} }_{\mathrm{solar}}$ = 0.872 and ${\overline{\varepsilon }}_{\mathrm{LWIR}}$ = 0.937 at a water content of 5 wt% can be achieved for RC at 6 wt% hBN and 2 wt% Al_2_O_3_ (Fig. 3c).

The thermogravimetric analysis (TGA) (Fig. 3d) results show that the solid content in the REC hydrogel was reduced to ~ 20% when the temperature was heated to 150 °C, indicating a high-water storage ratio of ~ 80%. Although the latent heat of water in the REC hydrogel was reduced from 2389 J g^−1^ in pure water to 2169 J g^−1^ (Fig. 3e), it was still far larger than the common phase change materials or RC materials. Due to functional groups and hydrogen bond formations, REC hydrogel conformally adheres to various surfaces with strong adhesive forces and excellent stability (Figs. 3f and S14).

### Indoor Heat & Mass Performance of REC Hydrogel

**Fig. 4:**
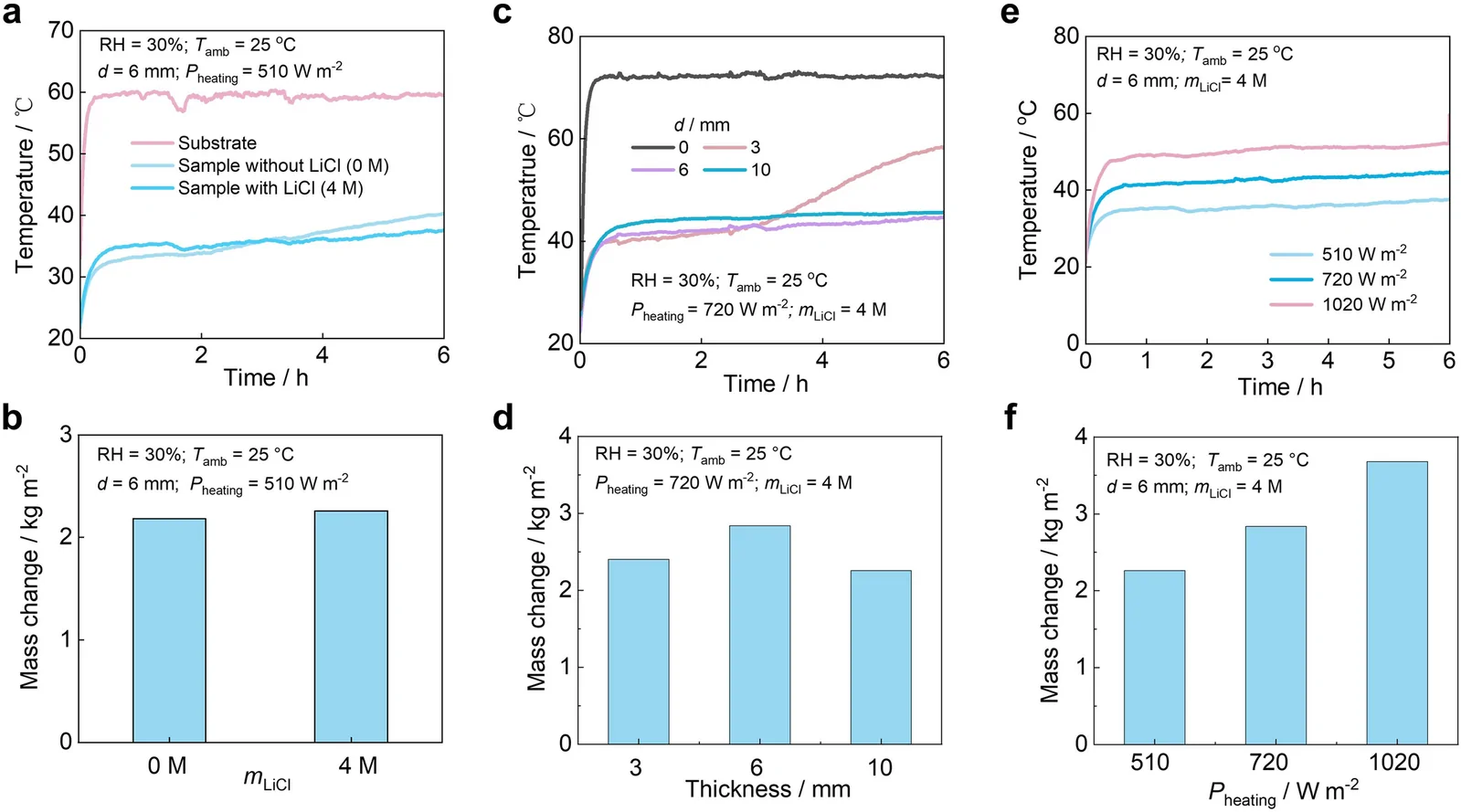
Indoor heat dissipation performance. **a** Temperature and **b** mass changes of REC hydrogel (with adsorbent LiCl via soaking in 4 M LiCl solution), pure REC hydrogel (without LiCl via soaking in pure water), and pure heater substrate (RH = 30%, *T*_amb_ = 25 °C, *P*_heating_ = 510 W m^−2^, and thickness *d* = 6 mm). **c** Temperature and **d** mass changes of REC hydrogel at different thicknesses *d* (RH = 30%, *T*_amb_ = 25 °C, *m*_LiCl_ = 4 M, and *P*_heating_ = 720 W m.^−2^). **e** Temperature and **f** mass changes of REC hydrogel at different heating powers *P*_heating_ (RH = 30%, *T*_amb_ = 25 °C, *d* = 6 mm, and *m*_LiCl_ = 4 M)

Based on the prepared REC hydrogel, the indoor heat and mass transfer performance was first studied to explore the heat dissipation and water capture performance. In the indoor environment, a constant temperature and humidity chamber was used to control *T*_amb_ and RH, as well as a heater that simulated the workload with different powers (Fig. S15). Details of the indoor experiments can be found in the experimental Section S2. For the indoor heat dissipation performance without sunlight, after coating the pure REC hydrogel on the heater, its working temperature quickly dropped from 59.7 to 40.3 °C under a heater power of 510 W m^−2^ for 6 h due to the latent heat release (Fig. 4a). When adding LiCl into the hydrogel, the designed REC hydrogel could achieve a relatively larger temperature rise initially due to the weakened EC, while its EC performance could maintain a relatively long period at the same water content. Hence, the designed REC hydrogel achieved the smallest temperature rise (37.6 °C) at the same total evaporation mass (Fig. 4b), which was 2.7 °C lower than the pure hydrogel. At the same water mass, the adsorbent could prolong evaporation time and maintain long-term cooling performance.

Water content was crucial for EC, which was also determined by hydrogel thickness *d,* while a large *d* would also increase thermal resistance *R*_t_, which was determined by ${R}_{\mathrm{t}}=d/\lambda$, where the thermal conductivity λ = 0.484 W m^−1^ K^−1^ measured by the Hot Disk. A small value of *d* (3 mm) led to water shortage in the long-term work, resulting in a poor heat dissipation performance with less evaporation mass (Fig. 4c). However, a large value of *d* (10 mm) increased the thermal resistance (0.021 m^2^ K W^−1^), leading to a relatively high working temperature with less evaporation mass (Fig. 4d). Hence, *d* = 6 mm was determined here to balance the water content and thermal resistance, which could maintain the relatively low working temperature under the same condition. Furthermore, when *P*_heating_ increased from 510 to 1020 W m^−2^, the working temperature increased from 37.6 to 59.7 °C, which was suppressed by the increased evaporation mass and radiation heat loss to the environment (Fig. 4e, f). These indoor results indicate that the designed REC hydrogel could achieve excellent heat dissipation performance due to multi-mode cooling strategies.

**Fig. 5:**
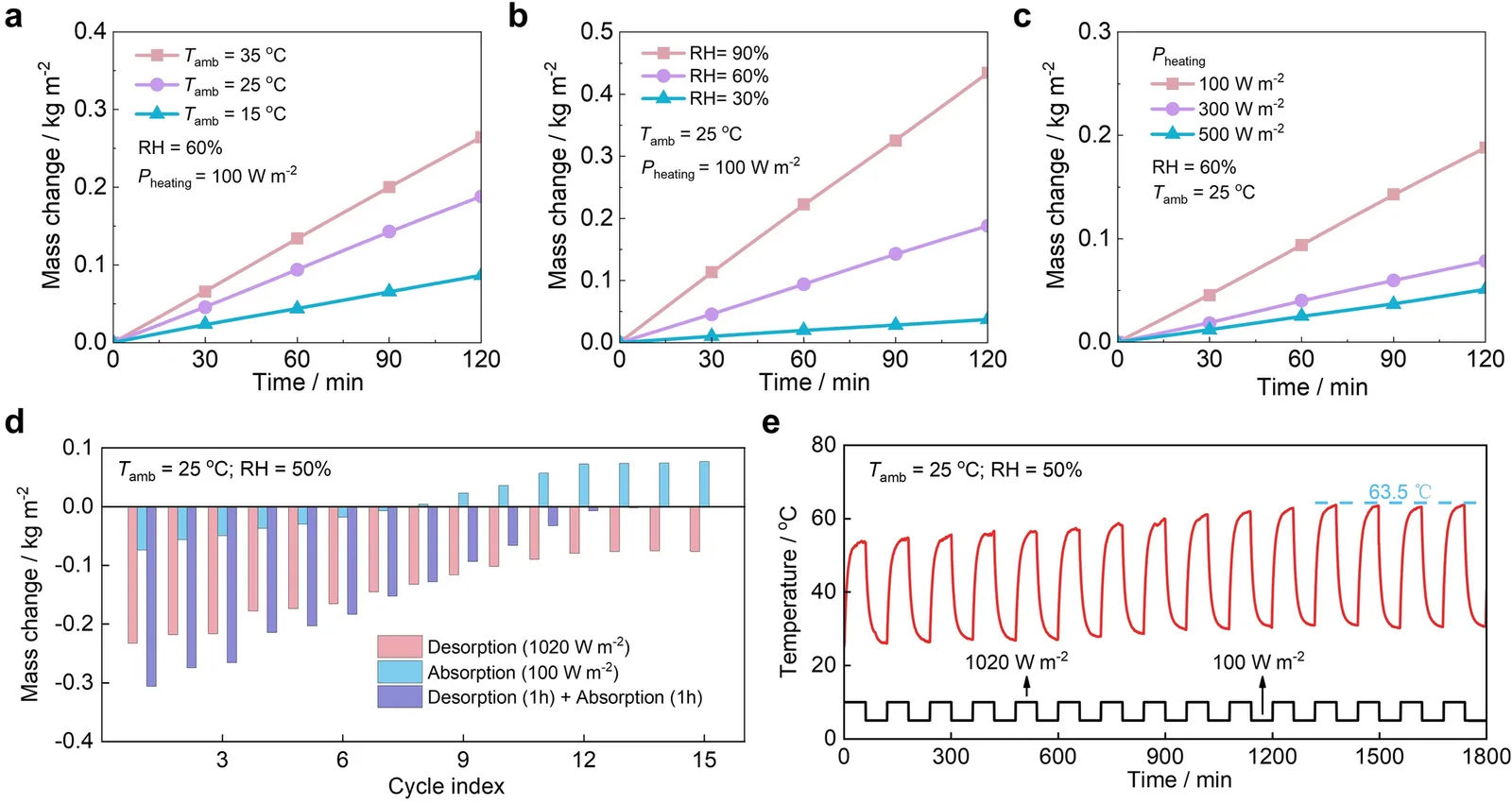
Indoor water capture and cycle performance. Mass change of REC hydrogel at different **a** *T*_amb_, **b** RH, and **c** *P*_heating_ (typical parameter: RH = 60%, *T*_amb_ = 25 °C, *P*_heating_ = 100 W m^−2^) in the nighttime with a low workload. **d** Mass and **e** temperature changes of REC hydrogel under 15 cycles of high workload (*P*_heating_ = 1020 W m^−2^ for 1 h) and low workload (*P*_heating_ = 100 W m^−2^ for 1 h) to evaluate its long-term stability, here RH = 50% and *T*_amb_ = 25 °C

In addition to water release for heat dissipation, water capture performance is another important factor for the water cycle, which was mainly determined by *T*_amb_, RH, and *P*_heating_. Under *T*_amb_ = 25 °C, RH = 90%, and *P*_heating_ = 100 W m^−2^, 62% of the saturated water capture was achieved at a water capture time of 12 h (Fig. S16). For the cycle of day and night, a period of 12 h for desorption and adsorption was difficult to achieve a saturated water content, which was beneficial to improve ${\overline{R} }_{\mathrm{solar}}$ at low water contents. Due to the heat release process as well as the heater in the water capture process, the working temperature is usually above ambient, and increasing *T*_amb_ can increase the water capture rate from 0.043 kg m^−2^ h^−1^ (*T*_amb_ = 15 °C) to 0.132 kg m^−2^ h^−1^ (*T*_amb_ = 35 °C) (Fig. 5a) due to the increase in saturated vapor pressure at the high *T*_amb_ (Fig. S3a), while the saturated water capture increased slightly from 4.34 to 4.50 kg m^−2^ (Fig. S17a).

When RH increased from 30% to 90%, the water capture rate increased from 0.019 to 0.217 kg m^−2^ h^−1^ (Fig. 5b), and the saturated water capture increased from 2.29 to 5.15 kg m^−2^ (Fig. S17b). In addition, a larger *P*_heating_ would suppress the water capture performance, which reduced from 0.094 (*P*_heating_ = 100 W m^−2^) to 0.026 (*P*_heating_ = 500 W m^−2^) kg m^−2^ h^−1^ in Fig. 5c. Hence, nighttime with high RH and low *P*_heating_ is beneficial for water capture performance. Finally, the indoor long-term stability was evaluated under the high workload (*P*_heating_ = 1020 W m^−2^ for 1 h) and low workload (*P*_heating_ = 100 W m^−2^ for 1 h) for 15 cycles. It can be found that the net mass change in a cycle would be zero (i.e., the balance between desorption and adsorption) as well as a stable working temperature (~ 63.5 °C) in Fig. 5d, e.

**Fig. 6:**
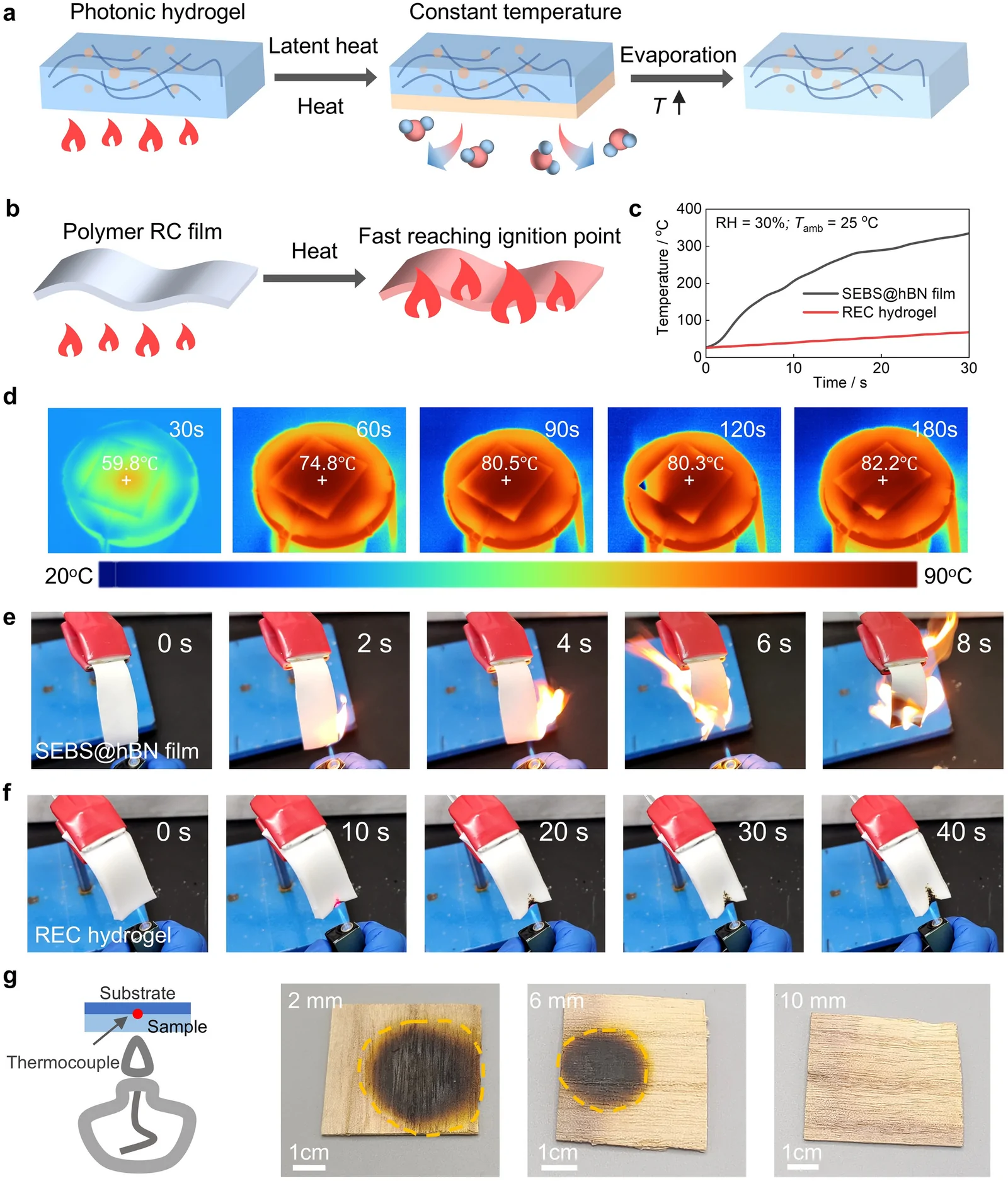
Flame retardancy performance of REC hydrogel. **a** Flame retardancy schematic diagram of REC hydrogel, as well as **b** the common polymer RC film, and **c** their real-time temperature change under the same flame of an alcohol lamp. **d** Infrared images of REC hydrogel (thickness 6 mm) under the same flame of an alcohol lamp. Optical images of **e** the common polymer RC film and **f** the REC hydrogel under the flame. **g** Schematic diagram and optical images of REC hydrogel with the wood substrate under the flame for 1200 s at different hydrogel thicknesses

For above-ambient heat dissipation, flame retardancy is also critical once the thermal runaway of the device occurs. The designed REC hydrogel improved flame retardancy by releasing the latent heat via EC without an obvious temperature increase, avoiding the fire risk (Fig. 6a). The traditional polymer RC was subjected to its operating temperature due to the low softening and ignition temperature, which further reduced cooling power and caused the fire (Fig. 6b). Under the same flame of the alcohol lamp, the temperature of SEBS@hBN RC film increased rapidly to 335 °C in 30 s while the temperature of REC hydrogel increased to ~ 68 °C when the sample was surrounded by fire (Fig. 6c). Detailed information about the SEBS@hBN RC film can be found in our previous work [47]. The thermal infrared images showed that the REC sample under the alcohol lamp maintained a temperature of ~ 82 °C for 180 s due to the latent heat of EC (Fig. 6d). Under the same flame, the SEBS@hBN RC film easily caught fire in several seconds (Fig. 6e), while the REC hydrogel had no obvious flames, although both had added the dielectric hBN particles (Fig. 6f). Furthermore, increasing the thickness of the REC hydrogel could enhance substrate protection by improving thermal resistance, as demonstrated with wood in Figs. 6g and S18. These results showed that the REC hydrogel had excellent above-ambient heat dissipation and flame retardancy.

### Outdoor Heat Dissipation Performance of REC Hydrogel

To evaluate the passive heat dissipation performance of REC hydrogel, outdoor experiments were conducted using a heater to simulate the workload (Fig. S19). Here, the pure heater substrate, SEBS@hBN RC film with high solar reflectance (~ 0.95) and thermal emittance (~ 0.93) [47], was used as the reference, whose spectral reflectance can be found in Fig. S20.

**Fig. 7:**
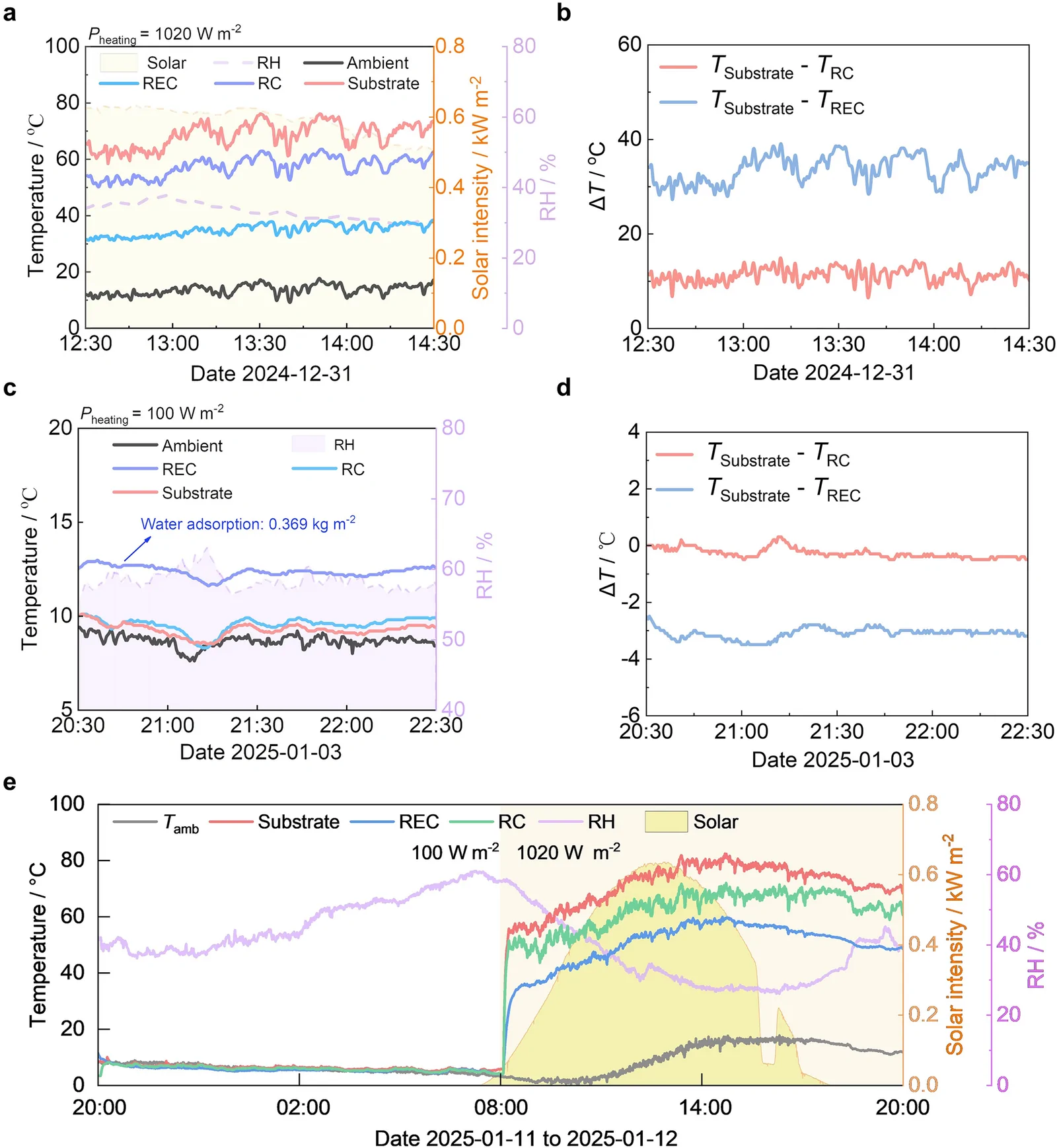
Outdoor heat dissipation performance of REC hydrogels. **a** Real-time temperatures of REC hydrogel, RC paint, and pure heater substrate, and **b** their temperature difference compared with the substrate in the daytime with the high workload (*P*_heating_ = 1020 W m^−2^) on 2024–12-31, including solar intensity, RH, and ambient temperature. **c** Real-time temperatures of REC hydrogel, RC paint, and heater substrate as the baseline and **d** their temperature difference compared with the substrate in the nighttime with the low workload (*P*_heating_ = 100 W m^−2^) on 2025–01-03. **e** Continuous all-day cycle performance of REC hydrogel (initial water content of 10%), RC paint, and pure heater substrate for three days from 2025–01-11 to 2025–01-12 (*P*_heating_ = 1020 W m^−2^ in the daytime with the high workload, and *P*_heating_ = 100 W m^−2^ in the nighttime with the low workload), including solar intensity, RH, and ambient temperature

In the daytime with a high workload (*P*_heating_ = 1020 W m^−2^), all samples achieved the above-ambient temperature due to the high *P*_heating_ (Fig. 7a). The pure substrate had the highest temperature rise due to the low solar reflectance (~ 0.48), followed by the RC film with high solar reflectance (~ 0.95), and the hydrogel achieved the lowest temperature rise due to REC. Compared with the substrate, the temperature drop of the REC hydrogel could be 22.4 °C lower than the RC film (11.2 vs. 33.6 °C) due to EC in Fig. 7b.

In the nighttime with a low workload (*P*_heating_ = 100 W m^−2^), due to the latent heat release in the water capture process, REC hydrogel achieved the highest temperature rise with a mass change of 0.369 kg m^−2^ in 2 h, substrate and RC film had the similar temperature rise at the same heating power due to the similar thermal emittance of ~ 0.93 (Fig. 7c). Compared with the substrate, the REC hydrogel had a temperature increase of ~ 3.1 °C while the RC film was only ~ 0.3 °C in Fig. 7d.

To further test the all-day heat dissipation performance of REC hydrogel with an initial dry state, continuous experiments are conducted in Fig. 7e. Here, a high workload with *P*_heating_ = 1020 W m^−2^ in the daytime and a low workload with *P*_heating_ = 100 W m^−2^ were assumed. It could be found that in the nighttime, all samples achieved a similar temperature to the ambient due to a low workload of 100 W m^−2^. In the daytime under the high workload of 1020 W m^−2^, all samples achieved the above-ambient working temperature, while the REC hydrogel was the smallest one, which was 20.9 °C lower than the substrate and 12.0 °C lower than the RC film (Table S1). When the workload was a constant value of 200 W m^−2^, water capture of the hydrogel in the nighttime with the high RH resulted in a higher working temperature than that of RC paint, while in the daytime the hydrogel achieved a smaller temperature increase than that of RC paint due to water release under sunlight with a low RH (Fig. S21), indicating that REC is a potential approach for above-ambient heat dissipation by an AWH-assisted passive water cycle, especially for the daytime heat dissipation under sunlight.

Further, the optical and SEM images indicated that there were no obvious salt crystals on the surface of the sample and substrate after drying the hydrogel (Fig. S22). To prevent salt ions from corroding the metal substrate, a protective layer between the substrate and hydrogel was suggested. In addition, the spectral reflectance and water capture performance of the hydrogel changed little after being placed outdoors for ten days (Fig. S23) and the total material cost of the hydrogel was 66.04 $ m^−2^ mm^−1^ (Table S2), further efforts can be done to improve its stability and reduce the cost in the practical applications. Finally, different hydrogels were prepared by replacing the polymer chain with other components, such as PVA, PAAm, and HPMC (Fig. S24). It can be found that the similar optical properties and water capture performances can be achieved, for example, solar reflectance and thermal emittance were 0.815/0.950, 0.845/0.937, and 0.833/0.956 for PVA, PAAm, and HPMC hydrogels at a water content of 10 wt%, and their water capture ability were 4.38, 3.01, and 3.84 kg m^−2^ for 16 h, which was slightly smaller than the designed hydrogel (0.851/0.951, and 5.15 kg m^−2^). Detailed radiative cooling and evaporation cooling performances of these hydrogels could be further optimized. In addition, previous works paid more attention to the bilayer structure in sub-ambient cooling (Table S3). The bilayer structure would increase the vapor transport resistance in water capture or release processes due to the top RC layer, resulting in a limited cooling performance in above-ambient heat dissipation. While the single layer was usually set at the backside of the solar cell for EC without RC, due to the limited solar reflectance. This designed all-in-one hydrogel shows excellent above-ambient heat dissipation and flame retardancy by coupling EC with RC, and a passive water recycle could be achieved by periodic workload and meteorological parameters.

## Conclusions

In summary, REC was successfully developed to achieve efficient above-ambient heat dissipation and enhanced flame retardancy through multi-mode cooling strategies. The all-in-one photonic hydrogel, composed of PDMAPS, LiCl, hBN nanoplates, and Al_2_O_3_, demonstrated versatile thermal management capabilities. During daytime periods characterized by high workloads, the integration of RC with EC significantly enhanced heat dissipation. Conversely, during nighttime conditions with lower workloads, RC-assisted adsorption effectively improved AWH, ensuring the availability of evaporative cooling resources for subsequent daytime use. In addition, the latent heat stored within the REC hydrogel effectively enhanced flame retardancy by absorbing heat without a corresponding temperature rise, thereby reducing fire risks.

The REC hydrogel exhibited radiative cooling properties at a water content of 5 wt%, with a high thermal emittance (${\overline{\varepsilon }}_{\mathrm{LWIR}}$ = 0.937) and solar reflectance (${\overline{R} }_{\mathrm{solar}}$ = 0.872). Outdoor experiments demonstrated that the REC hydrogel maintained a temperature of 12.0 °C lower than conventional RC film under identical conditions. The adsorbent LiCl component further extended cooling durations during daytime high workloads and significantly enhanced water capture during nighttime low workloads, facilitating a sustainable water cycle through repeated adsorption–desorption processes.

Furthermore, the REC hydrogel demonstrated exceptional flame retardancy. In comparative tests, the common SEBS@hBN RC film ignited quickly, with temperatures rising sharply to several hundred degrees Celsius, while the REC hydrogel remained flame-resistant, maintaining temperatures below 100 °C without visible flames. Overall, these results indicate that the REC hydrogel presents a promising solution for thermal management in outdoor devices, such as photovoltaic panels, battery power stations, and electronic devices, offering both superior heat dissipation performance and remarkable flame retardancy.

## Supplementary Information

Below is the link to the electronic supplementary material.

Supplementary file1 (DOCX 27457 KB)
