# Supplementary material for: Radiative Coupled Evaporation Cooling Hydrogel for Above-Ambient Heat Dissipation and Flame Retardancy
Source: Nanomicro Lett. 2025 Sep 1;18:50. doi: 10.1007/s40820-025-01903-0 (PMC12401836; doi:10.1007/s40820-025-01903-0)
Supplement: Supplementary file 1 — Supplementary file1 (DOCX 27457 KB) [file 40820_2025_1903_MOESM1_ESM.docx]

Supporting Information for

**Radiative Coupled Evaporation Cooling Hydrogel for Above-Ambient Heat Dissipation and Flame Retardancy**

Qin Ye^1,#^, Yimou Huang^1,#^, Baojian Yao^1,#^, Zhuo Chen^1^, Changming Shi^2^, Brian W. Sheldon^2^, Meijie Chen*

^1^ School of Energy Science and Engineering, Central South University, Changsha 430001, P. R. China

^2^ School of Engineering, Brown University, RI 02912, U.S.A.

^#^ Qin Ye, Yimou Huang, and Baojian Yao contributed equally to this work.

* Corresponding authors. E-mail: [chenmeijie@csu.edu.cn](mailto:chenmeijie@csu.edu.cn) (Meijie Chen)

**S1 Calculation model**

A theoretical model was built to calculate the temperature drop based on the energy balance law, which could also be found in our previous work [S1]. To simplify the calculation, a zero-dimensional model is considered here, where radiative coupled evaporative cooling and heating occur on the surface, which can be described as:

$P_{\text{REC}}=P_{\text{RC}}+P_{\text{EC}}=P_{\text{heating}}$ (S1)

where $P_{\text{RC}}$ was the radiative cooling power, $P_{\text{EC}}$ was the evaporation cooling power of the hydrogel, $P_{\text{REC}}$ was the radiative coupled evaporation cooling power, $P_{\text{heating}}$ is the heater's power.

***Heat transfer model***

$P_{\text{RC}}$ can be calculated as follows:

$P_{\text{RC}}=P_{\text{rad}}-P_{\text{sun}}-P_{\text{atm}}-P_{\text{nonrad}}$ (S2)

where $P_{\text{rad}}$ was the radiative power,$P_{\text{sun}}$ was the absorbed solar power,$P_{\text{atm}}$ was the absorbed radiative power from the atmospheric irradiation,$P_{\text{nonrad}}$ was the non-radiative heat transfer power between the hydrogel and the ambient.

$P_{rad}$ can be calculated as:

 (S3)

where $\varepsilon\left( \theta,\lambda\right)$ is the spectral directional emittance, $I_{BB}\left( T,\lambda\right)$ is the spectral intensity of a blackbody at the temperature *T*, which can be calculated by Planck’s law:

$I_{\text{BB}}\left( T,\lambda\right)=\frac{2hc^{2}}{\lambda^{5}\left( e^{\frac{hc}{\lambda kT}}-1 \right)}$ (S4)

where$h=6.626\times10^{-34}J s$is the Planck constant, $k=1.381\times10^{-23}J K^{-1}$is the Boltzmann constant, $c=3\times10^{8} m s^{-1}$is the speed of light in a vacuum, and $\lambda$ is the wavelength.

$P_{\text{sun}}$ can be expressed as：

$P_{\text{sun}}=\int_{\text{0.3μm}}^{\text{2.5μm}} \varepsilon\left(\theta,\lambda\right)I_{\text{solar}}\left(\lambda\right)d\lambda$ (S5)

where $I_{\text{solar}}\left( \lambda\right)$ is the spectral solar intensity at AM 1.5.

$P_{\text{atm}}$ can be expressed as:

 (S6)

where $\varepsilon_{\text{atm}}\left( \theta,\lambda\right)$ is atmospheric emittance as a function of incident zenith angle *θ* and wavelength, which can be approximated by:

$\varepsilon_{\text{atm}}\left( \theta,\lambda\right)=1-[\tau\left( \lambda\right)]^{1/\cos\theta}$ (S7)

where $\tau\left( \lambda\right)$ is the atmospheric transmittance in the zenith direction, and it depends on the ambient temperature $T_{\text{atm}}$ and relative humidity $\varphi$. Here we first converted the parameter pair ($T_{\text{atm}}$,$\varphi$) to the precipitable water vapor (PWV) [S2] and then input PWV to MODTRAN to achieve the atmospheric transmittance [S3].

$P_{\text{nonrad}}$ was defined as:

$P_{\text{nonrad}}=h_{c}\left( T_{\text{amb}}-T \right)$ (S8)

where $h_{\text{c}}$ is the non-radiative heat transfer coefficient, including the conductive and convective processes.

***Mass transfer model***

$P_{\text{EC}}$ can be calculated based on the mass change rate：

$\Delta m=\frac{P_{\text{EC}}}{H_{v}\left( T \right)}$ (S9)

where $\Delta m$ is the vapor mass flux, which can be calculated in the mass transfer model, $H_{v}\left( T \right)$ is vaporization latent heat at the temperature *T*.

$H_{v}\left( T \right)$ is phase change latent heat at the temperature *T*, which can be calculated by [S4]：

$H_{v}\left( T \right)=1918460(\frac{T}{T-33.91})^{2}$ (S10)

$\Delta m$ is the vapor mass flux, which can be calculated by the water vapor concentration difference between the ambient and the surface,$\Delta m$ can be written as [S5]:

$\Delta m=g_{m,H_{2}O}\left( \hat{m}_{H_{2}O,\text{ambient}}-\hat{m}_{H_{2}O,\text{surface}} \right)$ (S11)

where $g_{m,\text{H}_{2}\text{O}}$ was the mass transfer coefficient of the water vapor, $\hat{m}_{\text{H}_{2}\text{O},\text{ambient}}$ and $\hat{m}_{\text{H}_{2}\text{O},\text{surface}}$ were the vapor concentration of water vapor in the ambient and near the hydrogel surface, respectively, which can be calculated based on the Ideal Gas Law:

$\hat{m}_{H_{2}O}=\frac{RH\times P_{H_{2}O}M_{H_{2}O}}{P_{\infty}M_{\text{air}}}$ (S12)

where $P_{\infty}$ was the atmospheric pressure,$M_{\text{H}_{2}\text{O}}$ and $M_{\text{air}}$ were the molecular mass of water and air, respectively, $P_{\text{H}_{2}\text{O}}$ was the saturation vapor pressure in units of [10^-3^ bar], which can be determined by ^6^:

$P_{H_{2}O}=10^{A-B/\left( t+C \right)}$ (S13)

For *T* in 0 − 60 °C, *A* = 8.10765, *B* = 1750.286, *C* = 235.

**Supplementary Figures**


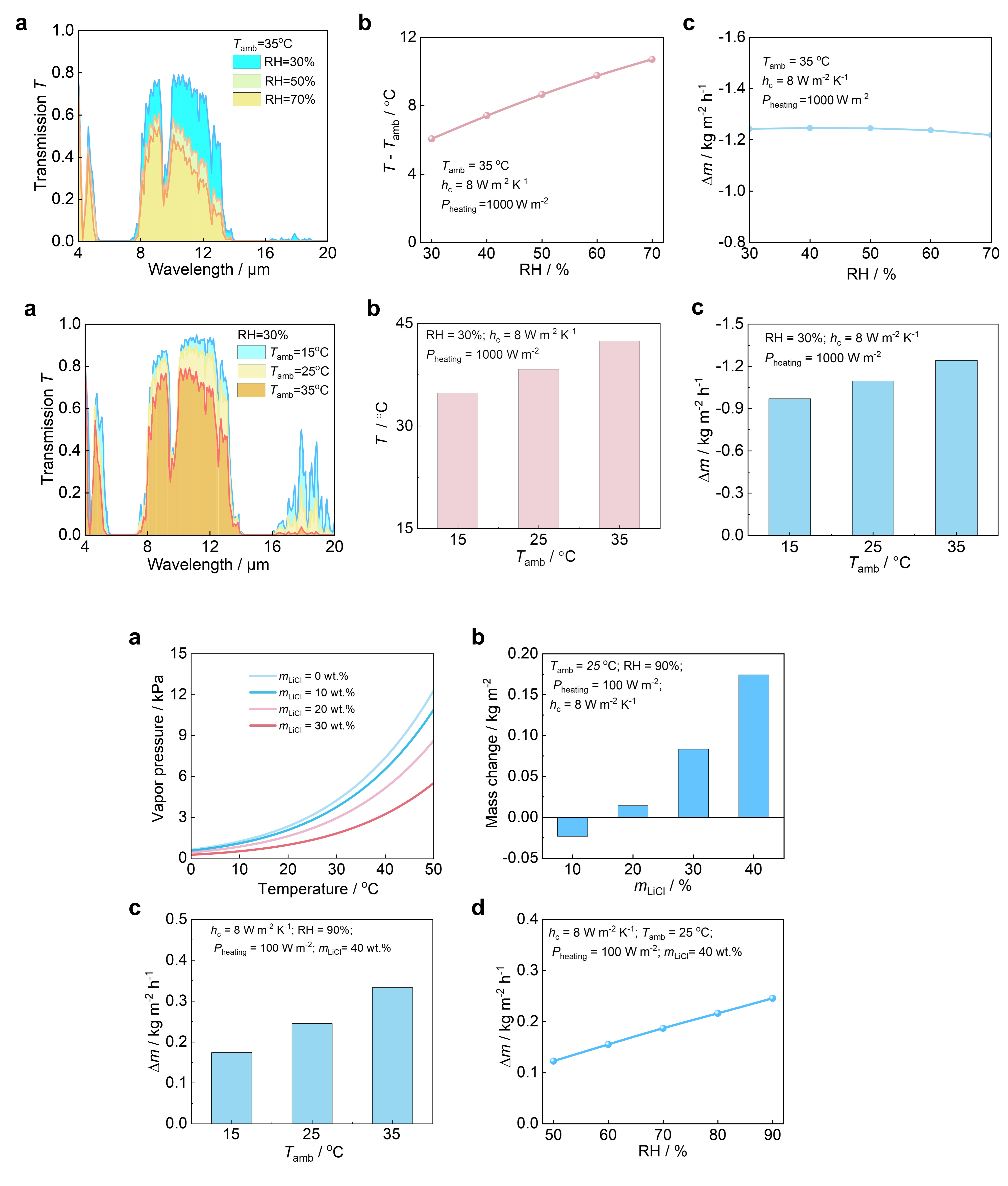


**Fig. S1** Effect of RH on the heat dissipation performance. **a** Atmospheric transmittance spectra, **b** temperature, and **c** mass changes of the ideal REC at different RH


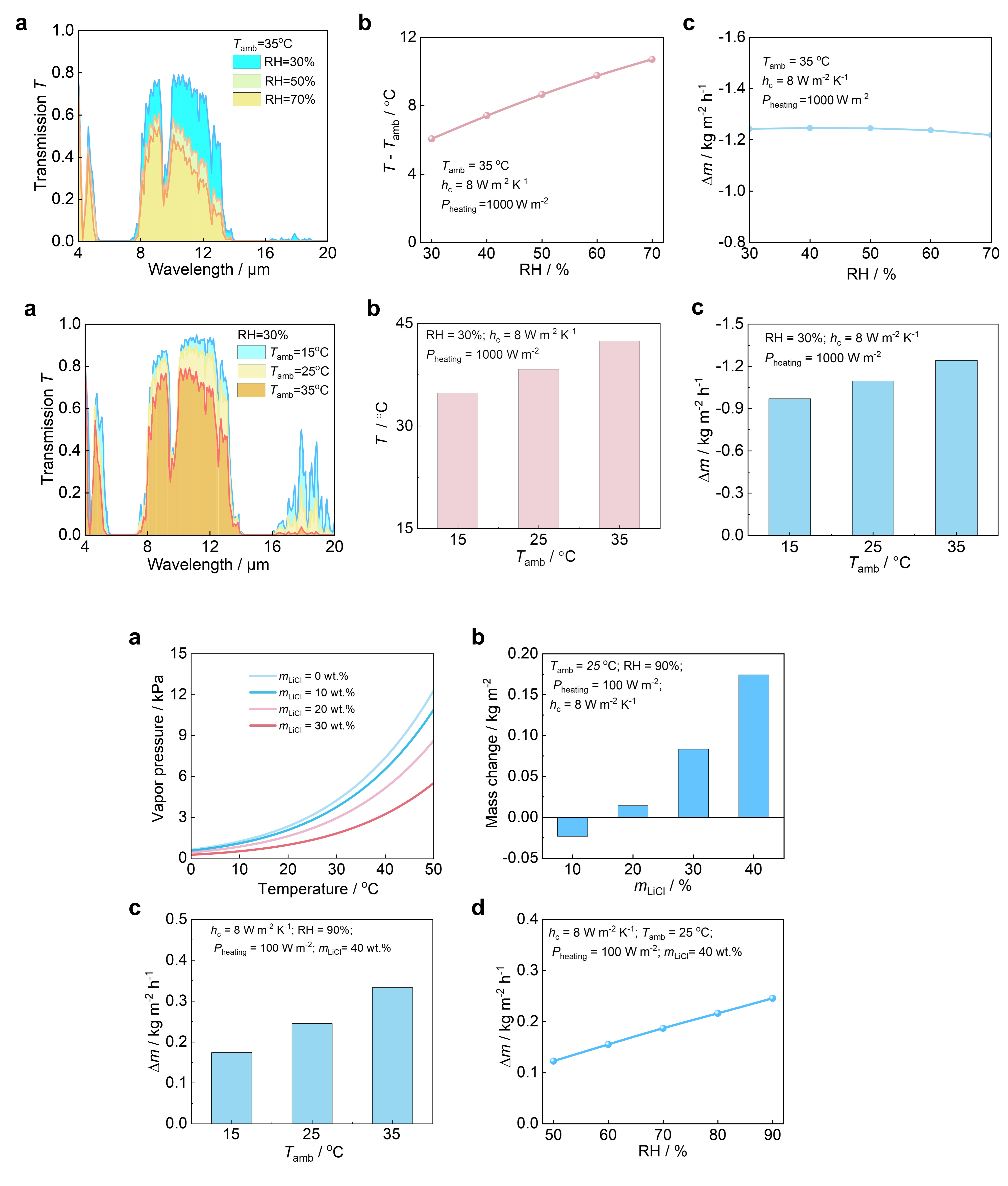


**Fig. S2** Effect of *T*_amb_ on the heat dissipation performance. **a** Atmospheric transmittance spectra, **b** temperature, and **c** mass changes of the ideal REC at different *T*_amb_


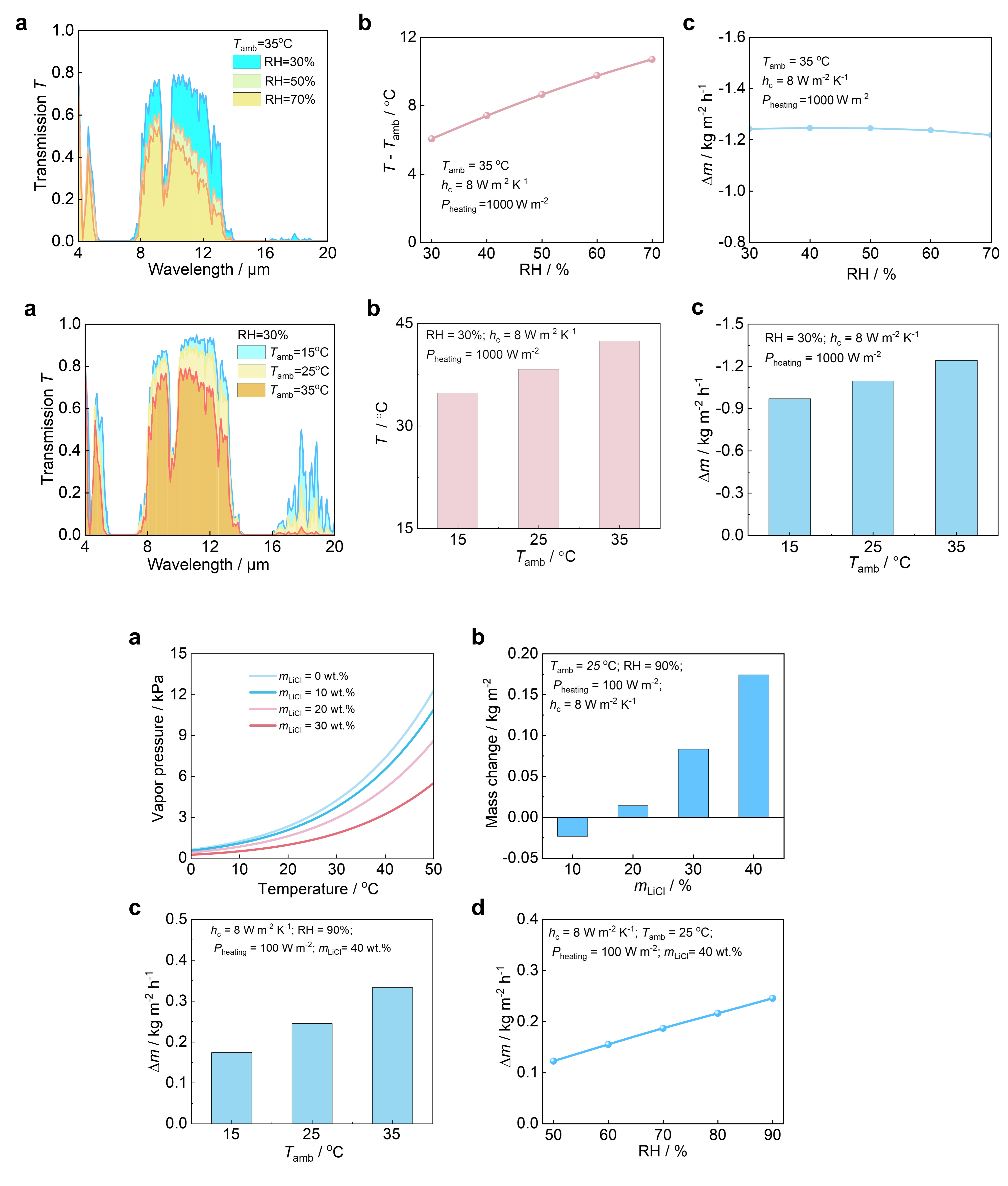


**Fig. S3** Water capture performance calculation. **a** Vapor pressure at different *m*_LiCl_. Mass change of the sample at different **b** *m*_LiCl_, **c** *T*_amb_, and **d** RH


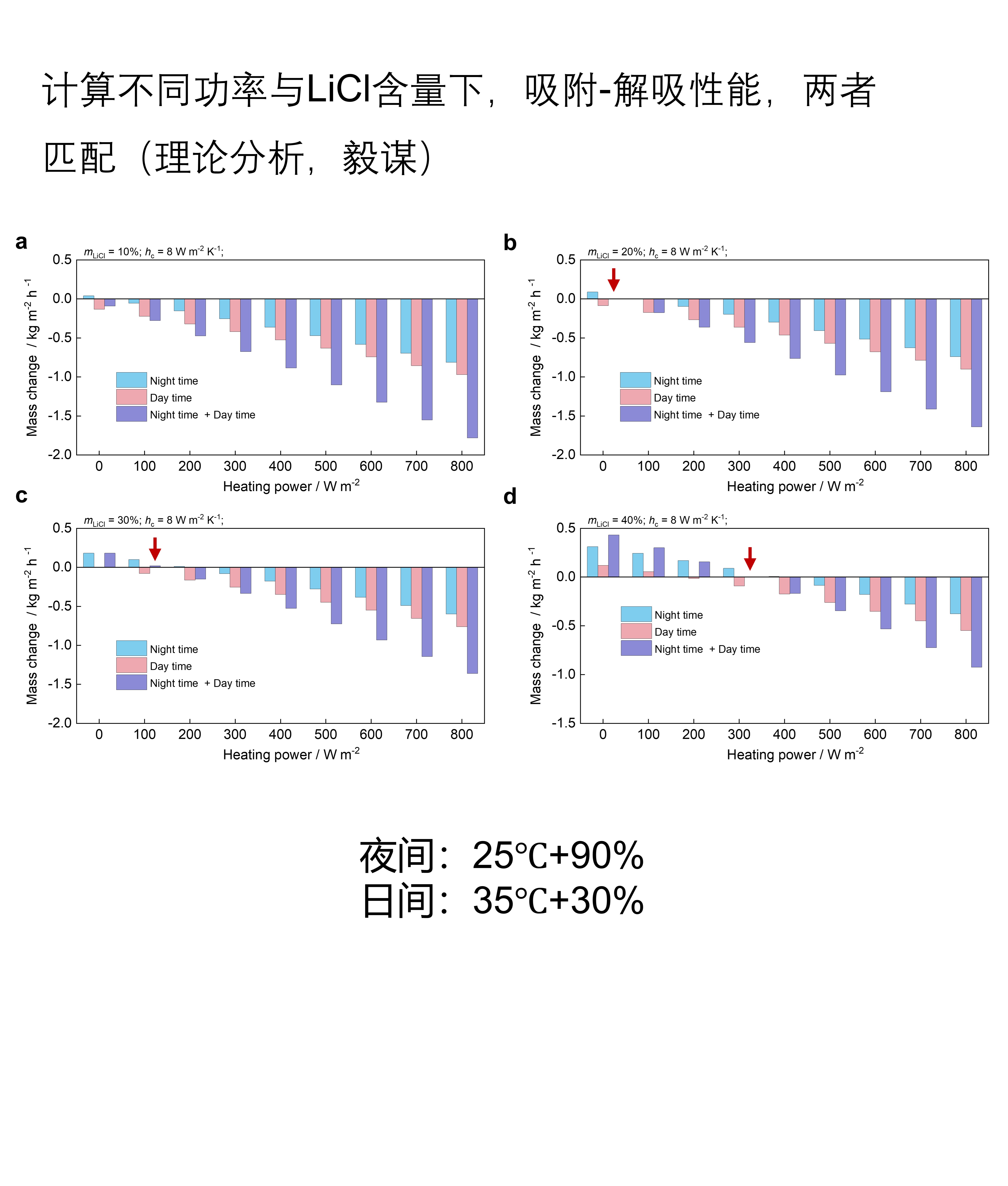


**Fig. S4** Calculated water change in the nighttime (*T*_amb_ = 25 ^o^C, RH = 90%), daytime (*T*_amb_ = 35 ^o^C, RH = 30%), and all day at different heating powers based on different LiCl mass fractions (**a** for 10%, **b** for 20%, **c** for 30%, and **d** for 40%)


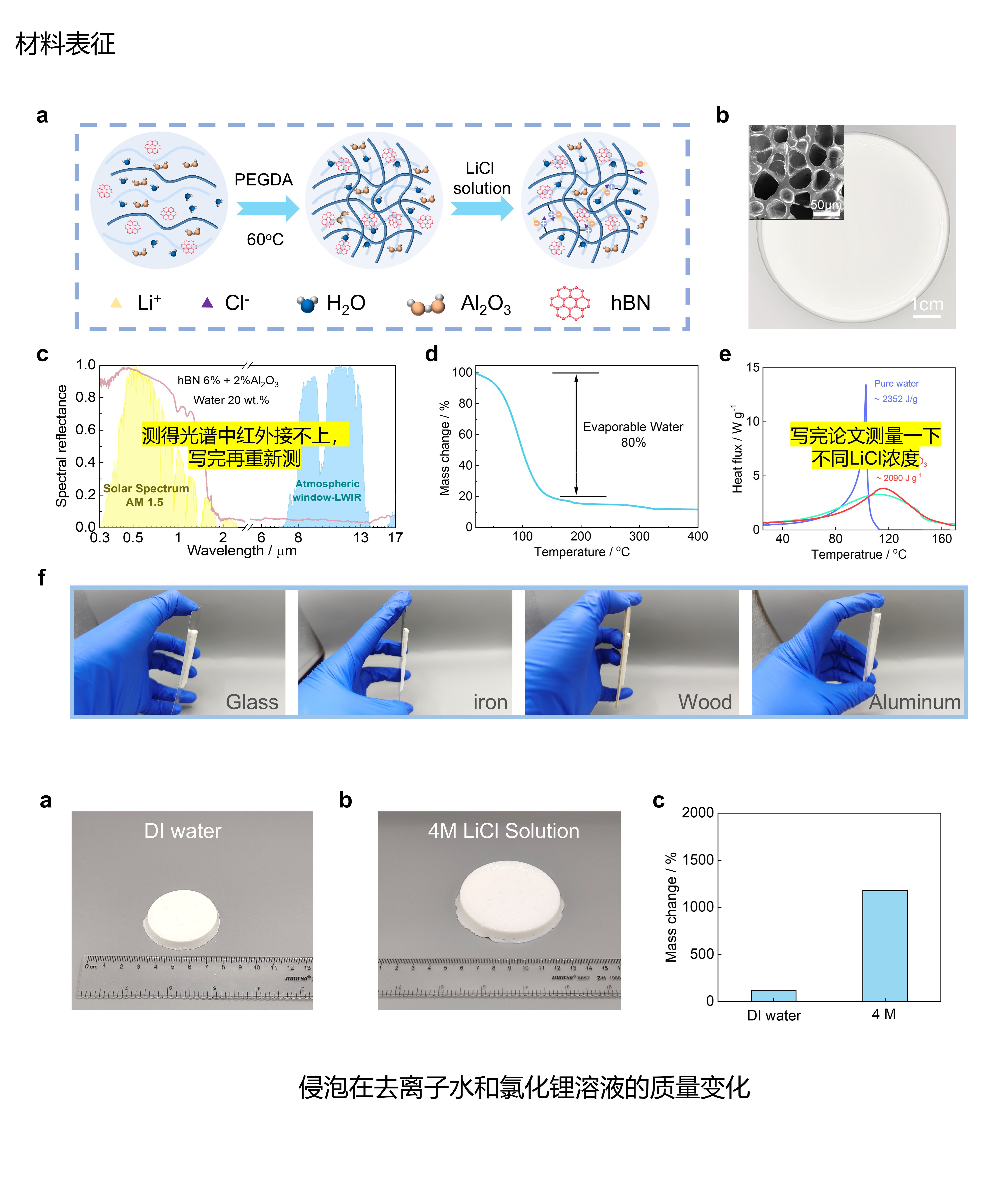


**Fig. S5** Optical images of the hydrogel after soaking **a** pure water, **b** 4 M LiCl solution, and **c** their mass changes


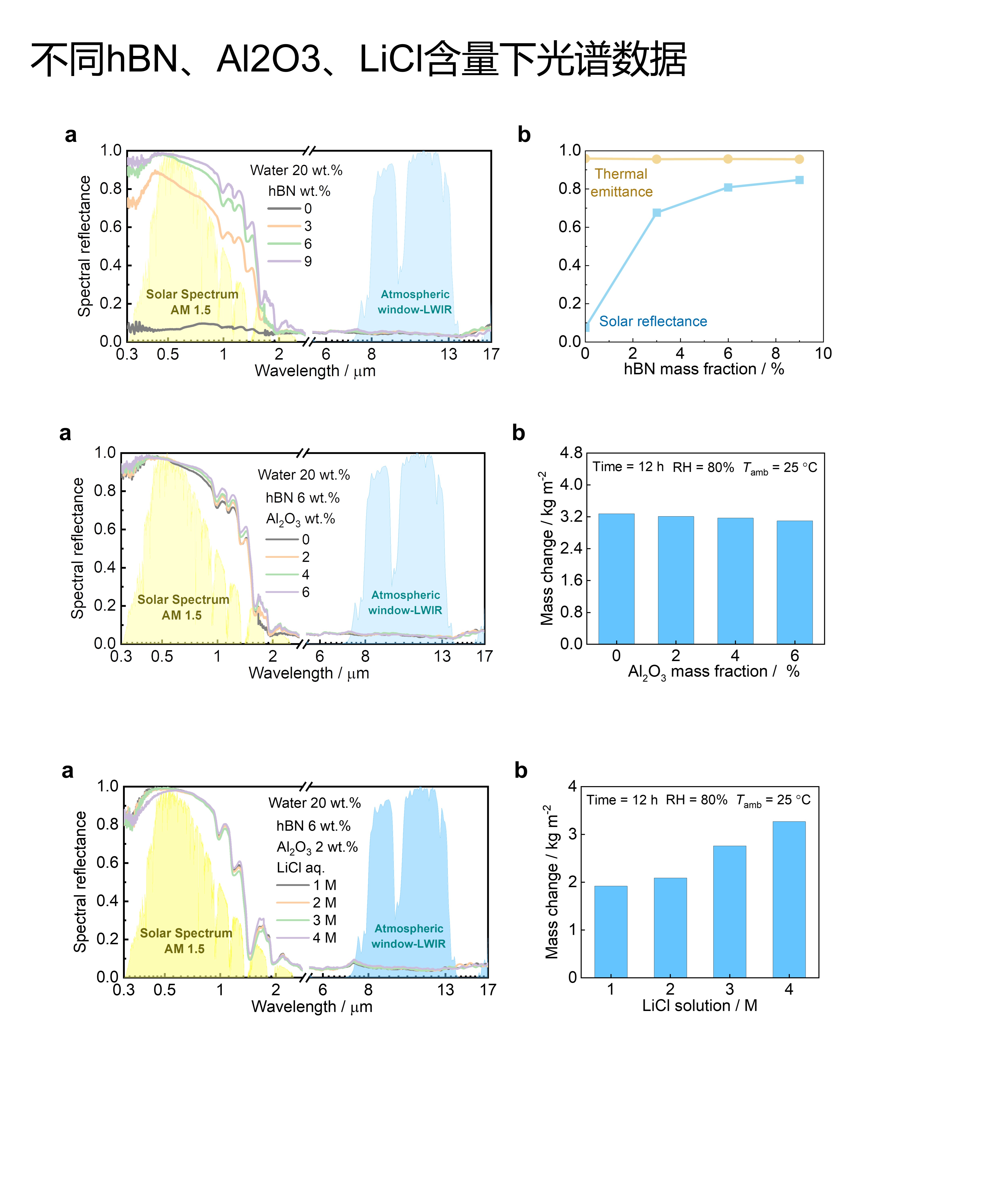


**Fig. S6** **a** Spectral reflectance and **b** water capture performance of photonic hydrogels after soaking in different LiCl aqueous solutions


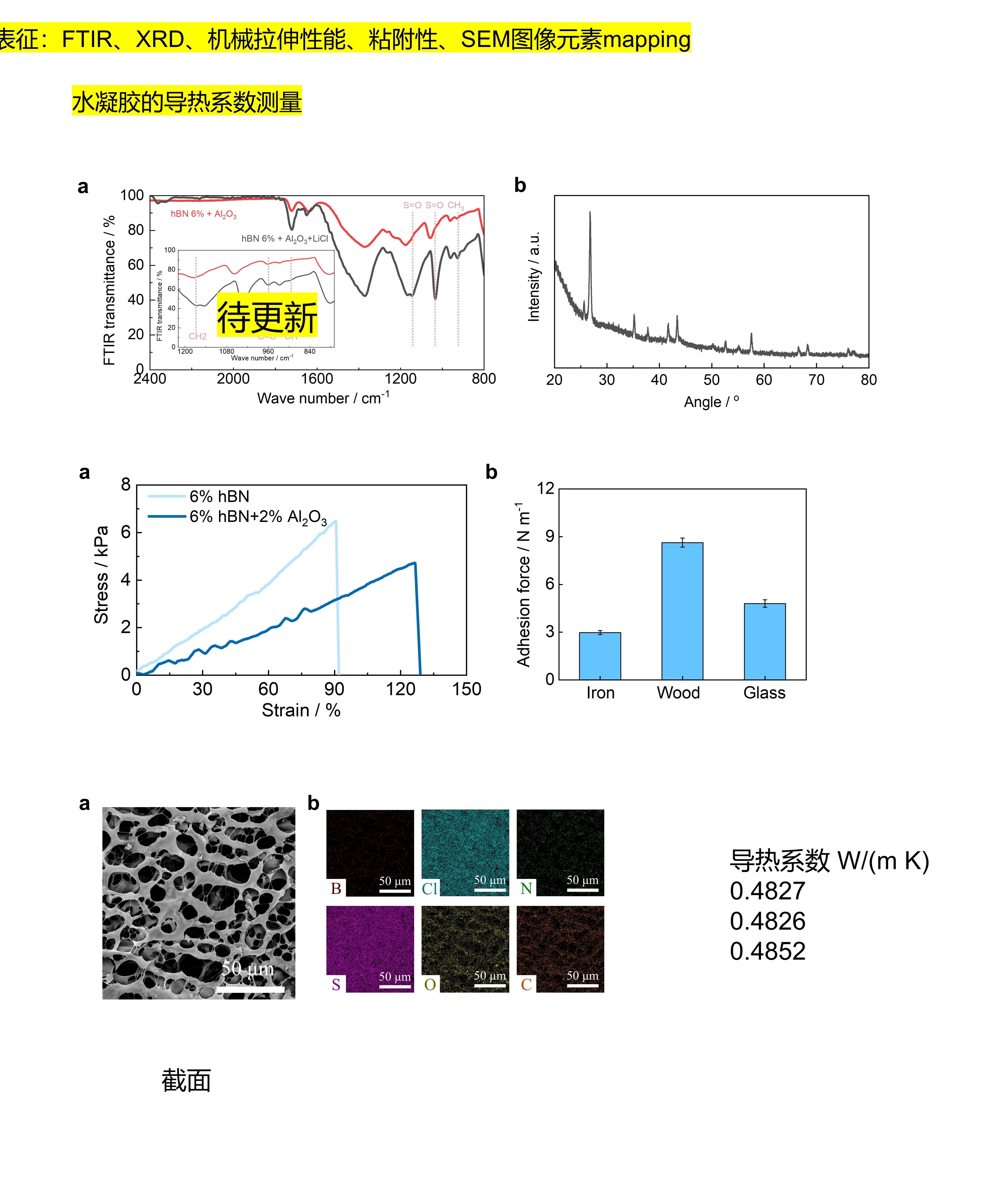


**Fig. S7** **a** SEM and **b** EDS mapping images of the photonic hydrogel at the cross-section


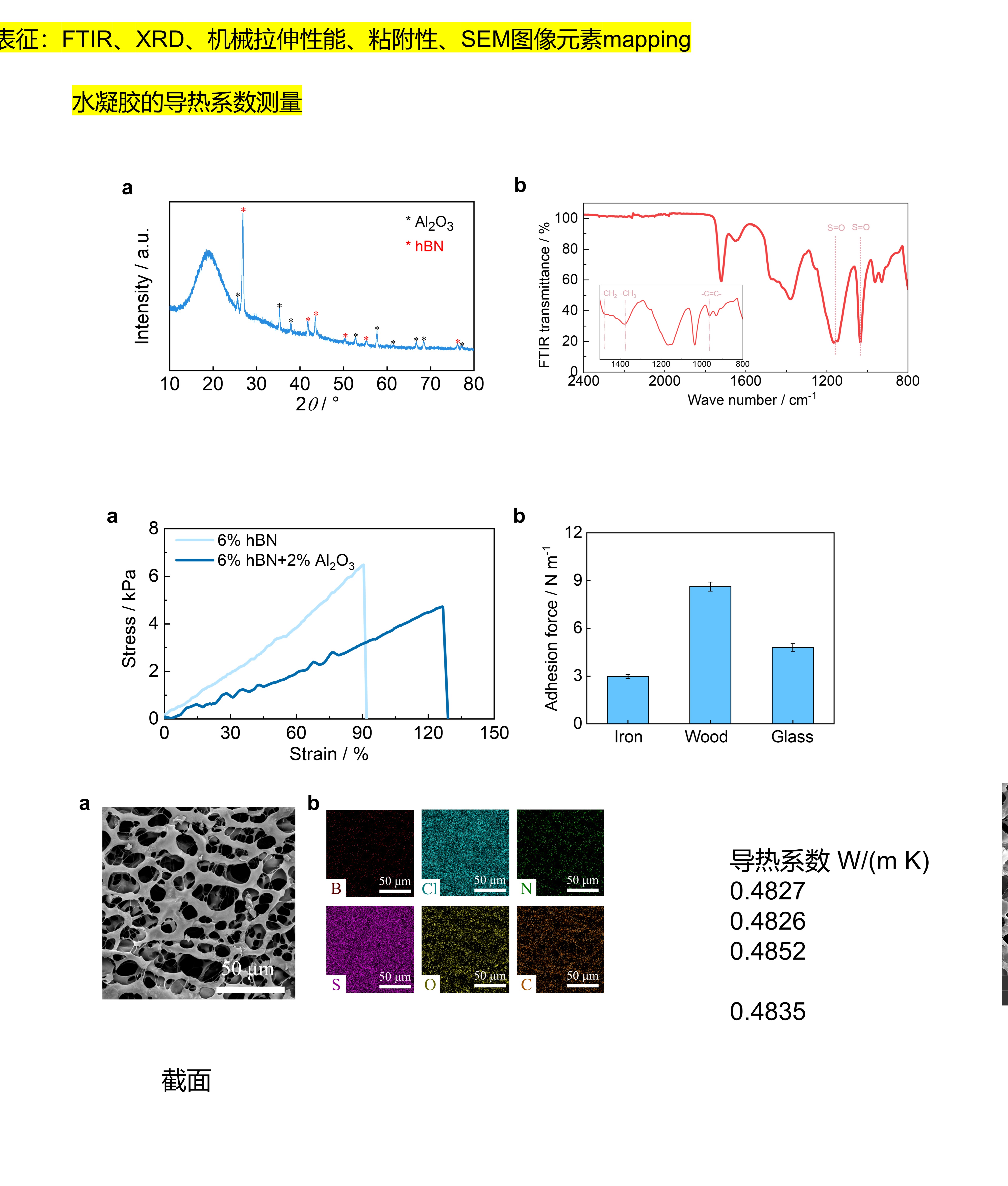


**Fig. S8** **a** X-ray diffraction results (XRD) and **b** Fourier-transform infrared (FTIR) spectroscopy results of the photonic hydrogel


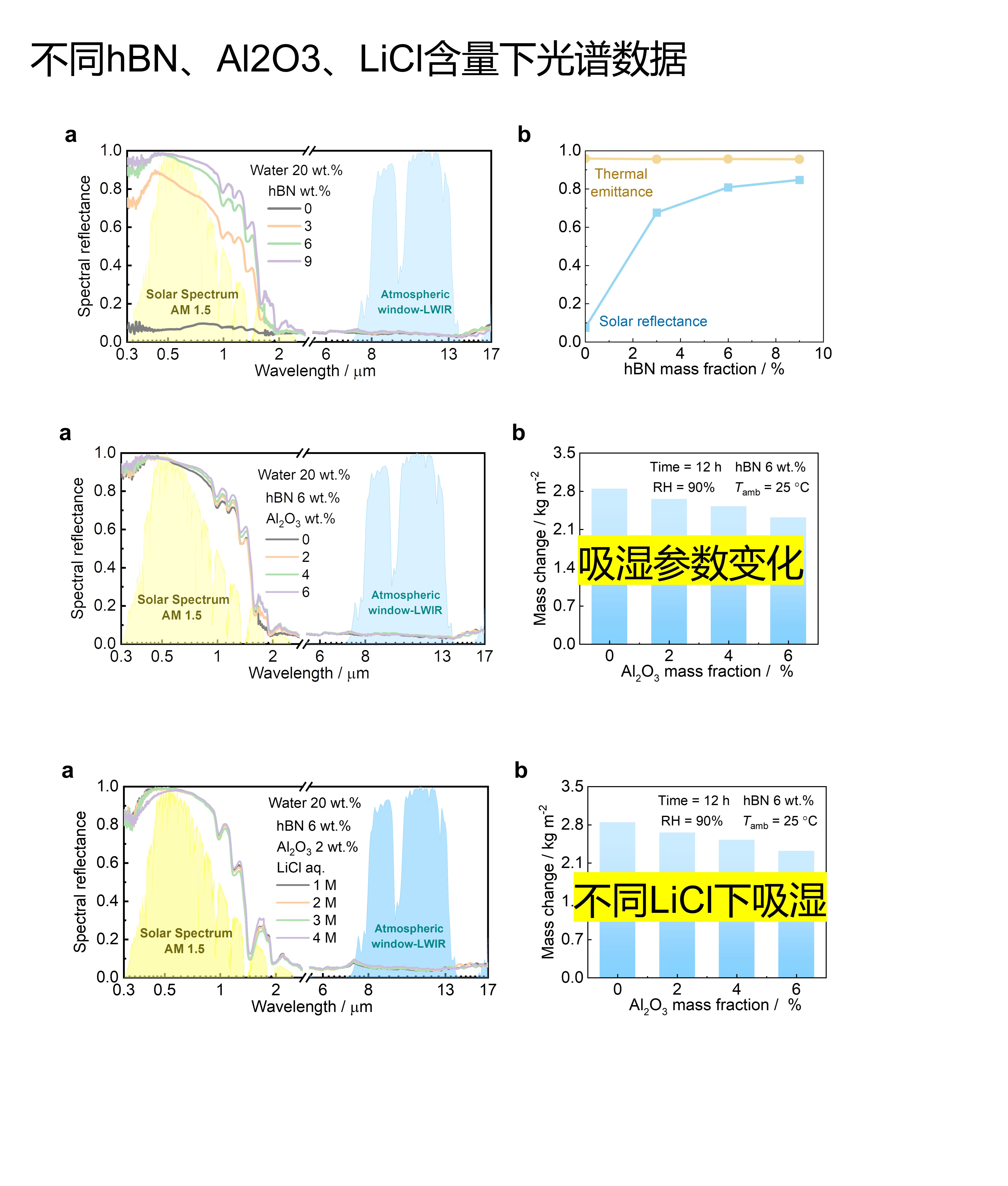


**Fig. S9** **a** Spectral reflectance of hydrogels, **b** average solar reflectance ($\bar{R}_{\mathrm{solar}}$) and mid-infrared thermal emittance ($\bar{\varepsilon}_{\mathrm{LWIR}}$) with different hBN mass fractions (*m*_hBN_) at a water content of 20 wt.%


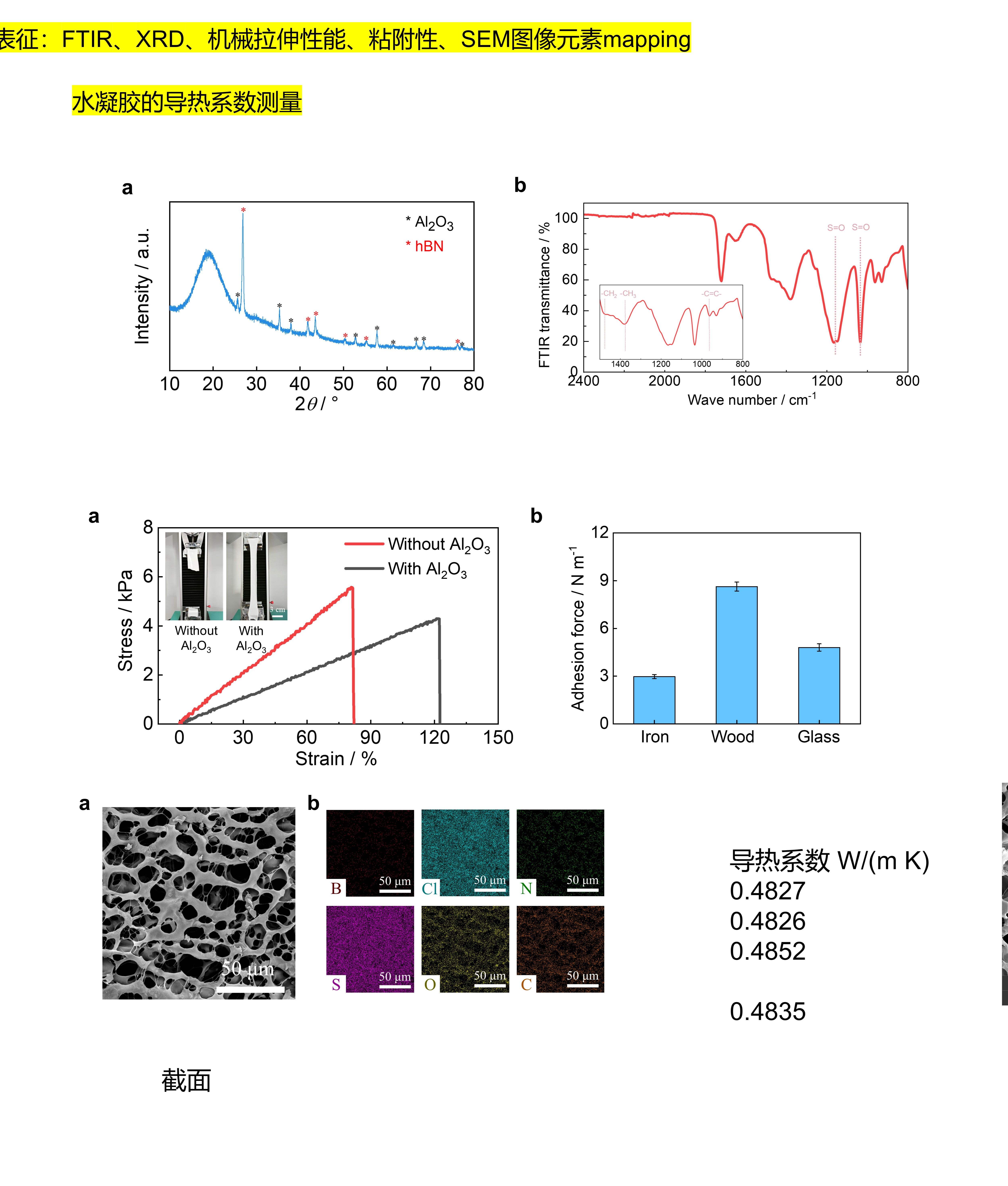


**Fig. S10** Tensile strain of the photonic hydrogel with Al_2_O_3_ or without Al_2_O_3_


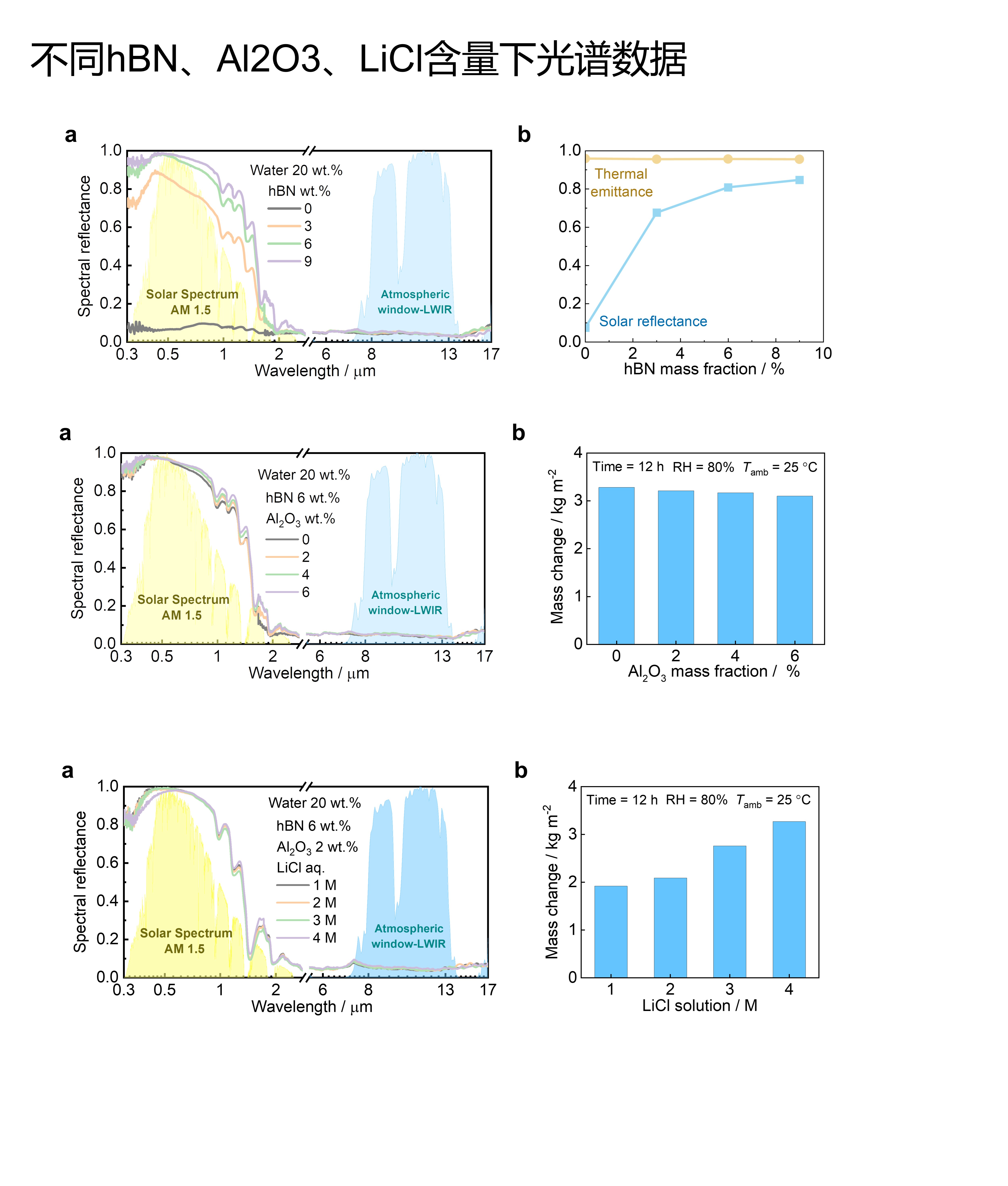


**Fig. S11** **a** Spectral reflectance and **b** water capture performance of photonic hydrogels with different Al_2_O_3_ mass fractions


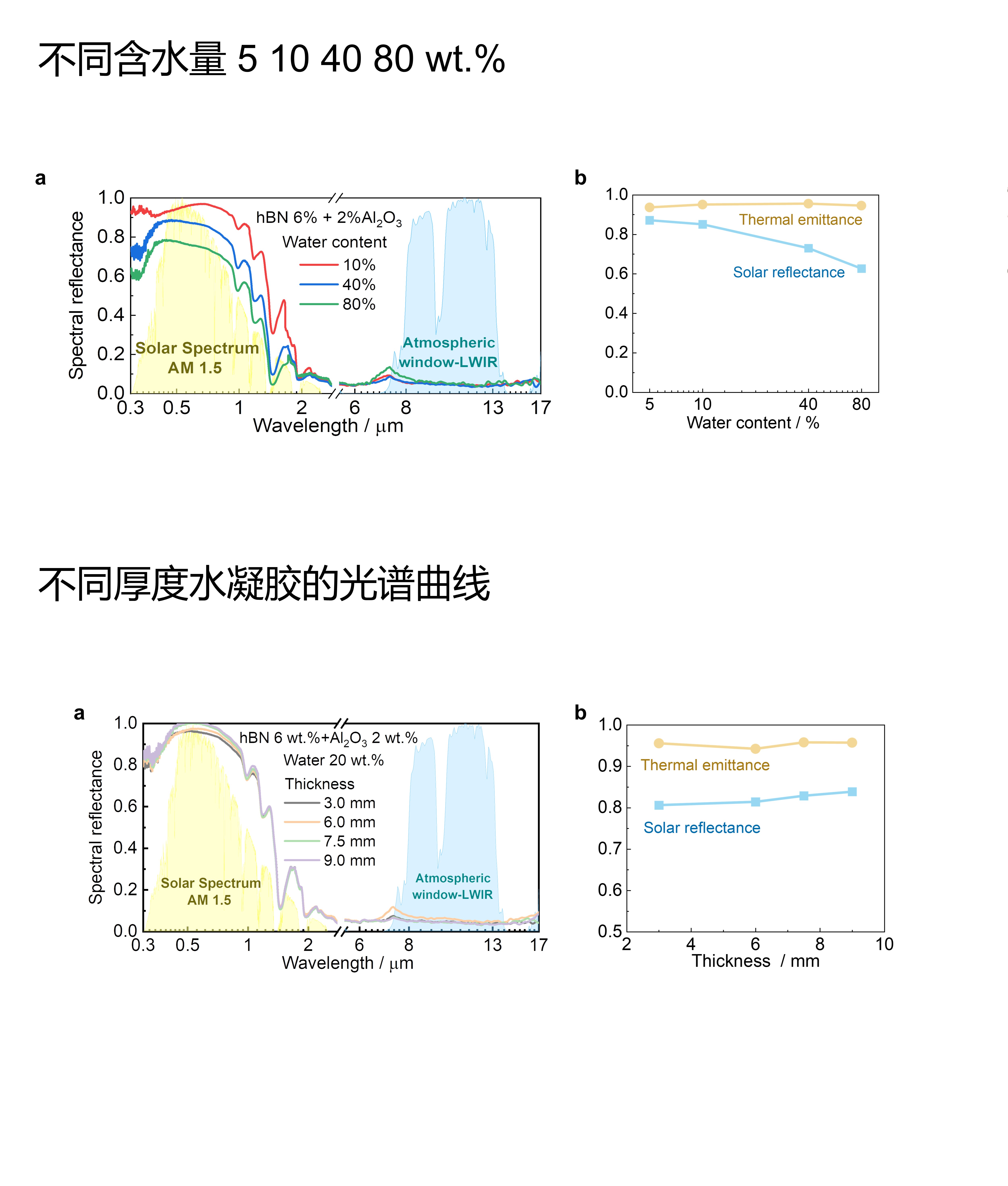


**Fig. S12** Spectral reflectance of hydrogels with different thicknesses at a water content of 20 wt.%. Backgrounds are the normalized solar irradiance (yellow) and atmospheric transmittance windows (cyan)


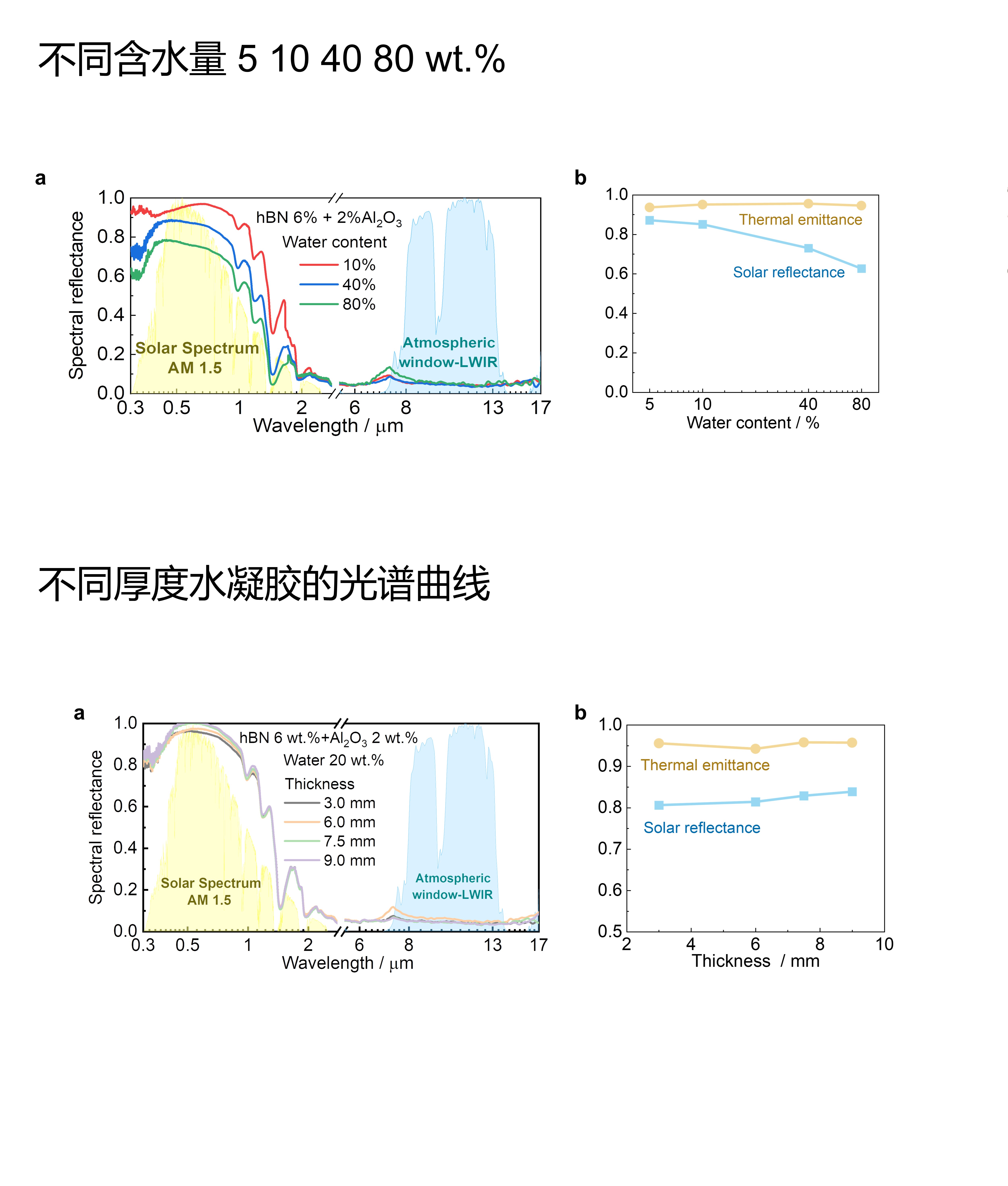


**Fig. S13** Spectral reflectance of hydrogels with different water contents. Backgrounds are the normalized solar irradiance (yellow) and atmospheric transmittance windows (cyan)


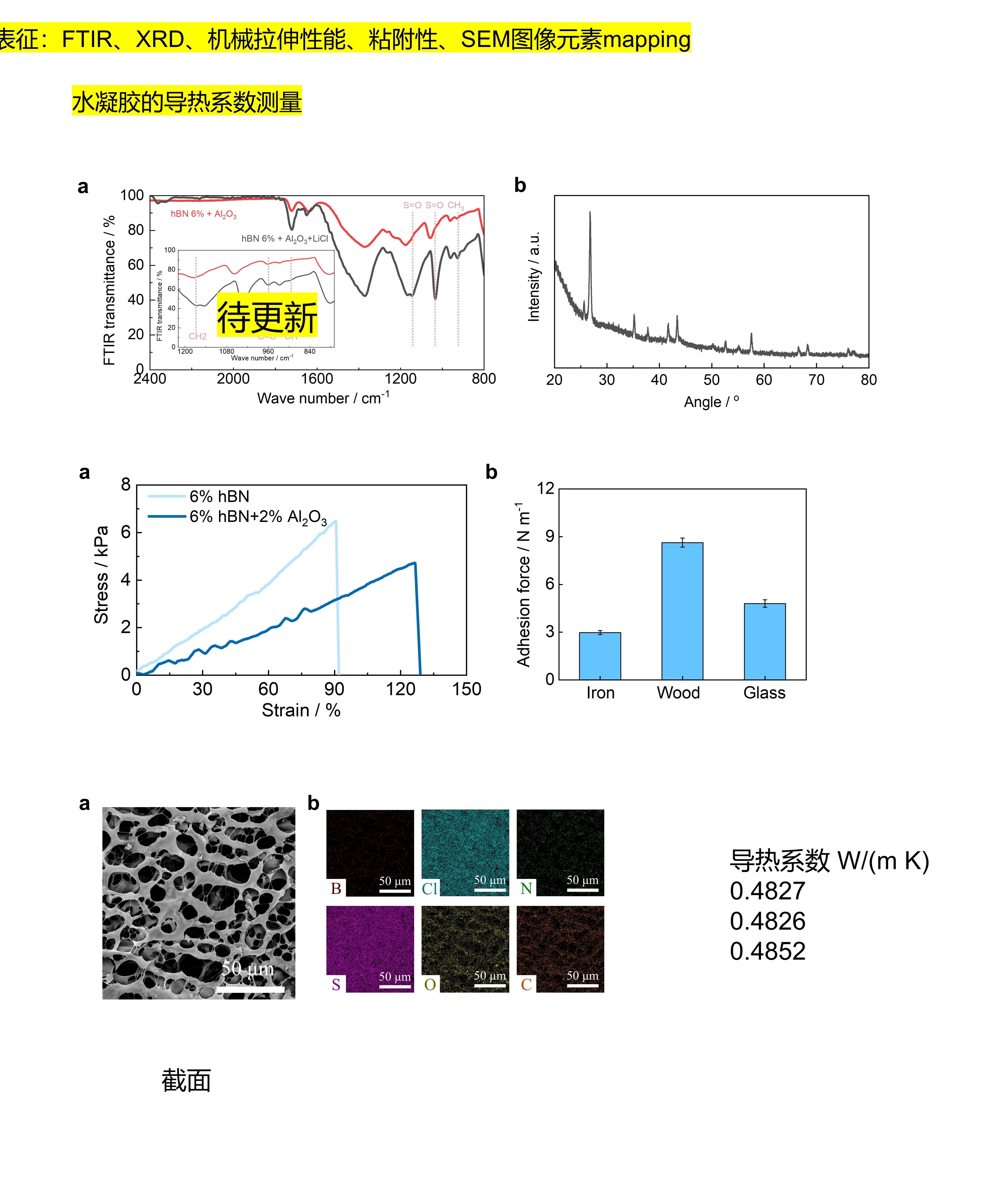


**Fig. S14** 90º peel test for interfacial toughness measurement on different substrates


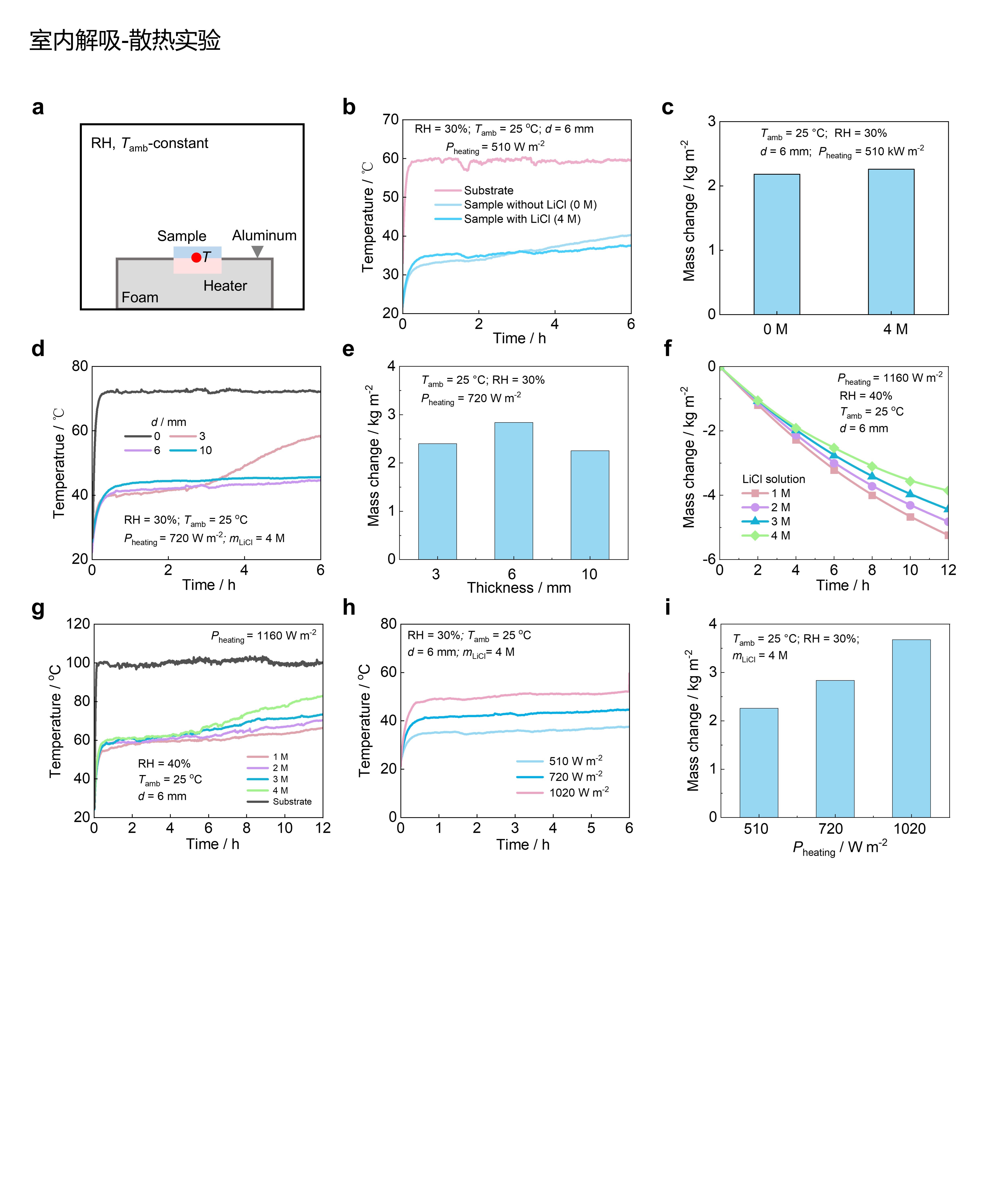


**Fig. S15** Schematic diagram of the indoor experimental setup in the constant temperature and humidity chamber


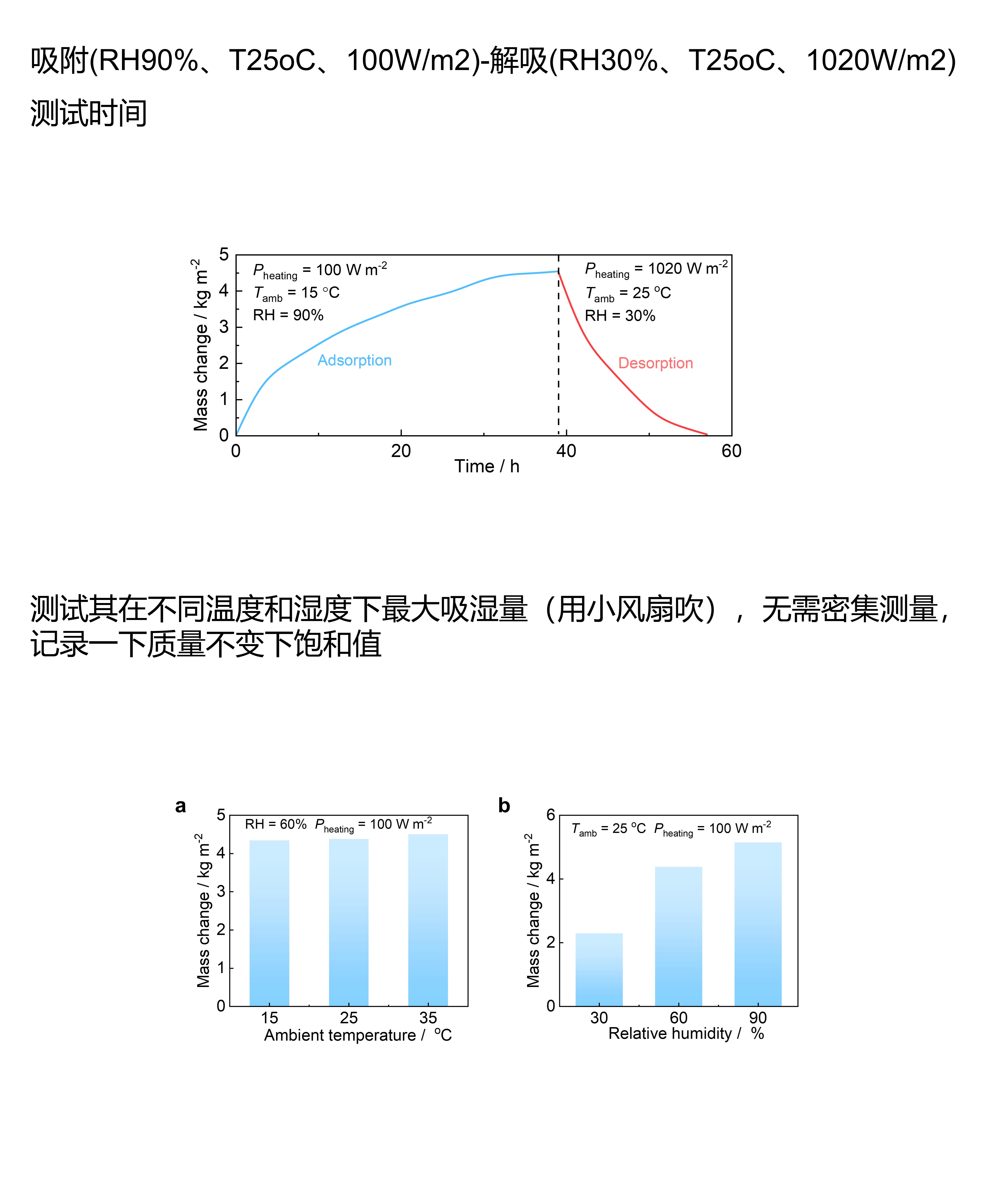


**Fig. S16** Water capture and release cycle of the photonic hydrogel (initial water content of 15 %). Here, water capture under RH = 90%, and *T*_amb_ = 15 ^o^C with a low workload of 100 W m^-2^, and water release under RH = 30%, and *T*_amb_ = 25 ^o^C with a high workload of 1020 W m^-2^


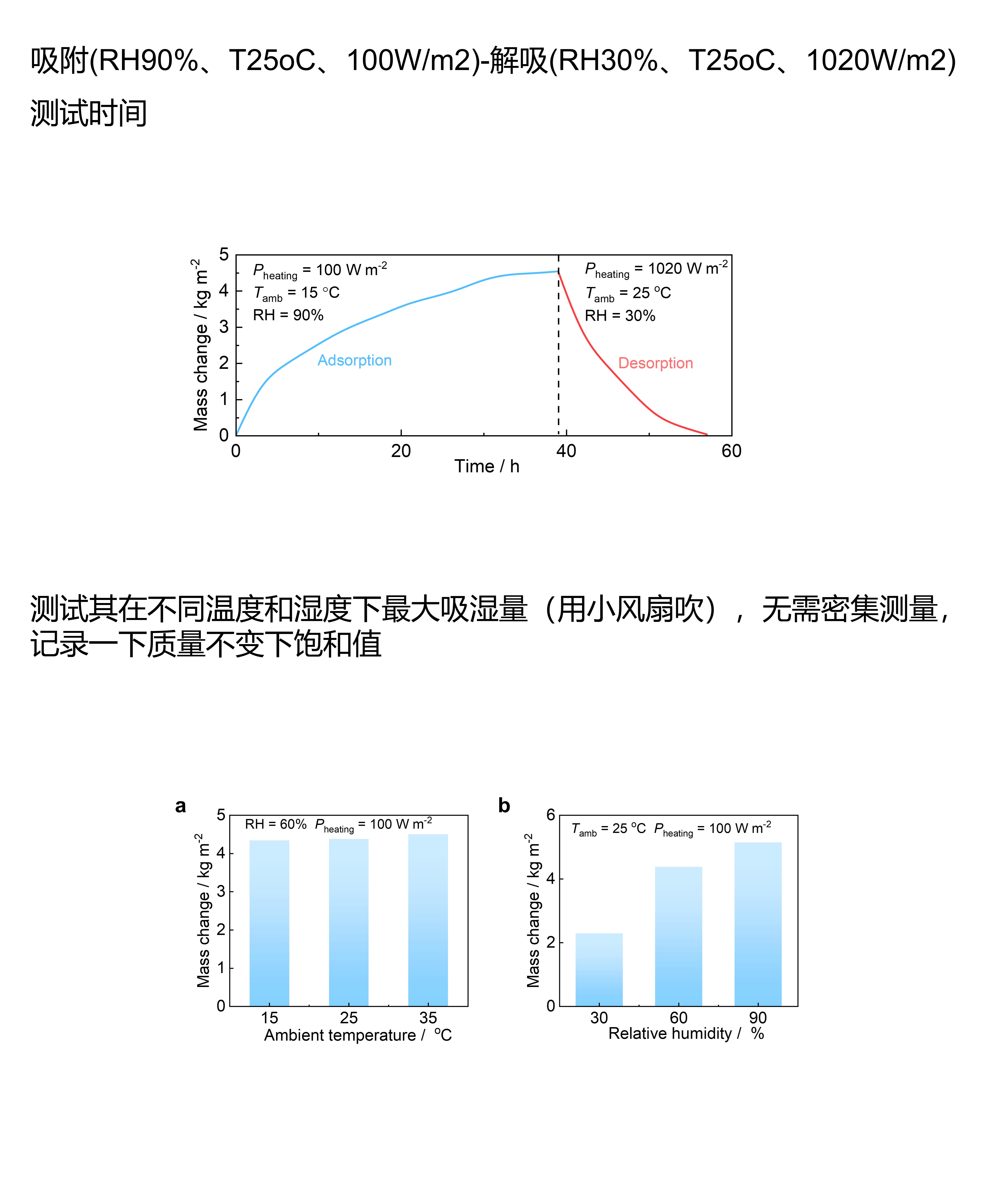


**Fig. S17** Saturated mass change of REC hydrogel at different **a** *T*_amb_, and **b** RH in the nighttime with a low workload


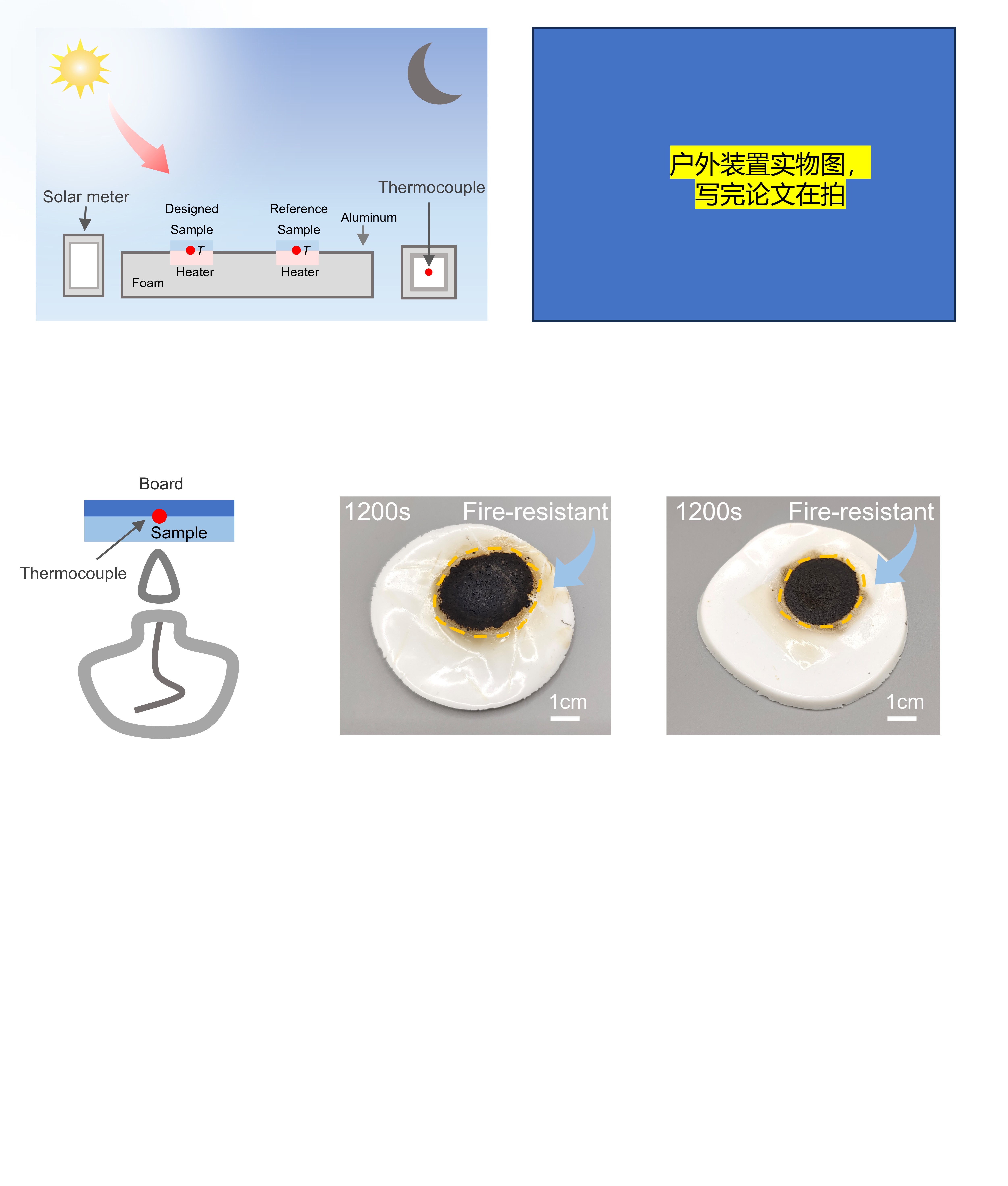


**Fig. S18** Schematic diagram of flame retardancy experiment setup and its optical images at 1200s


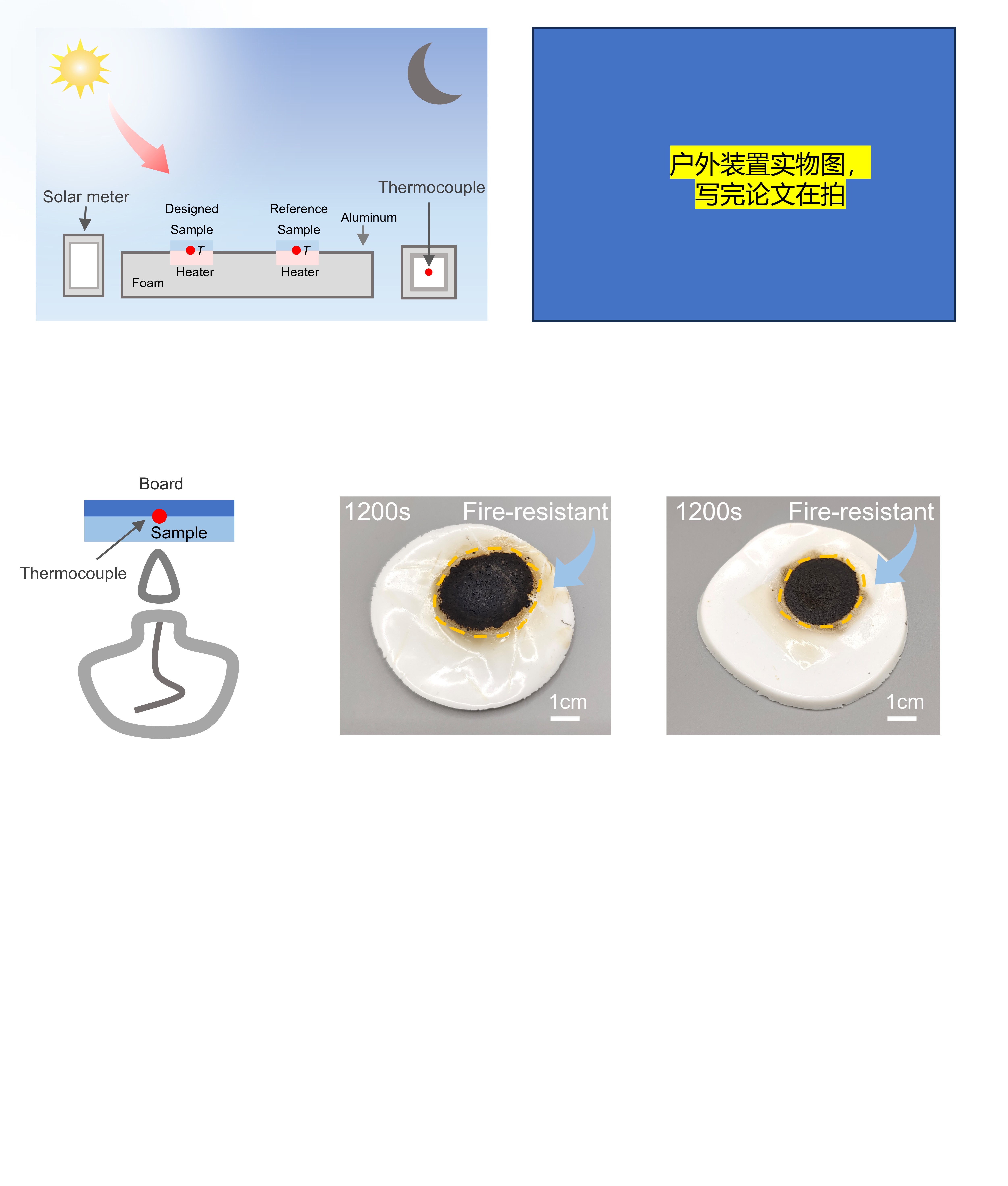


**Fig. S19** Schematic diagram of the outdoor experimental setup


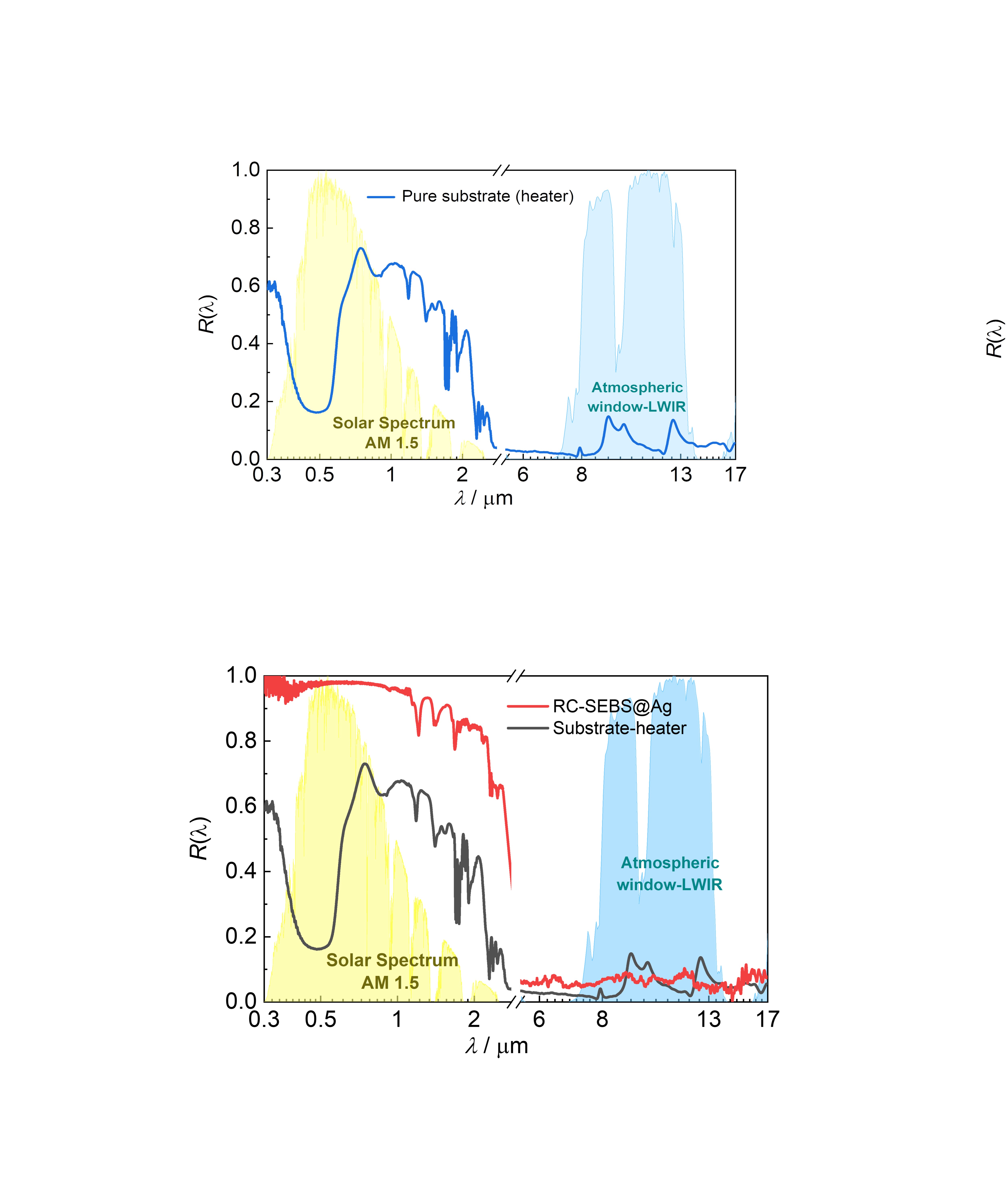


**Fig. S20** Spectral reflectance of the reference substrate heater and SEBS@hBN radiative cooler from Ref. 7


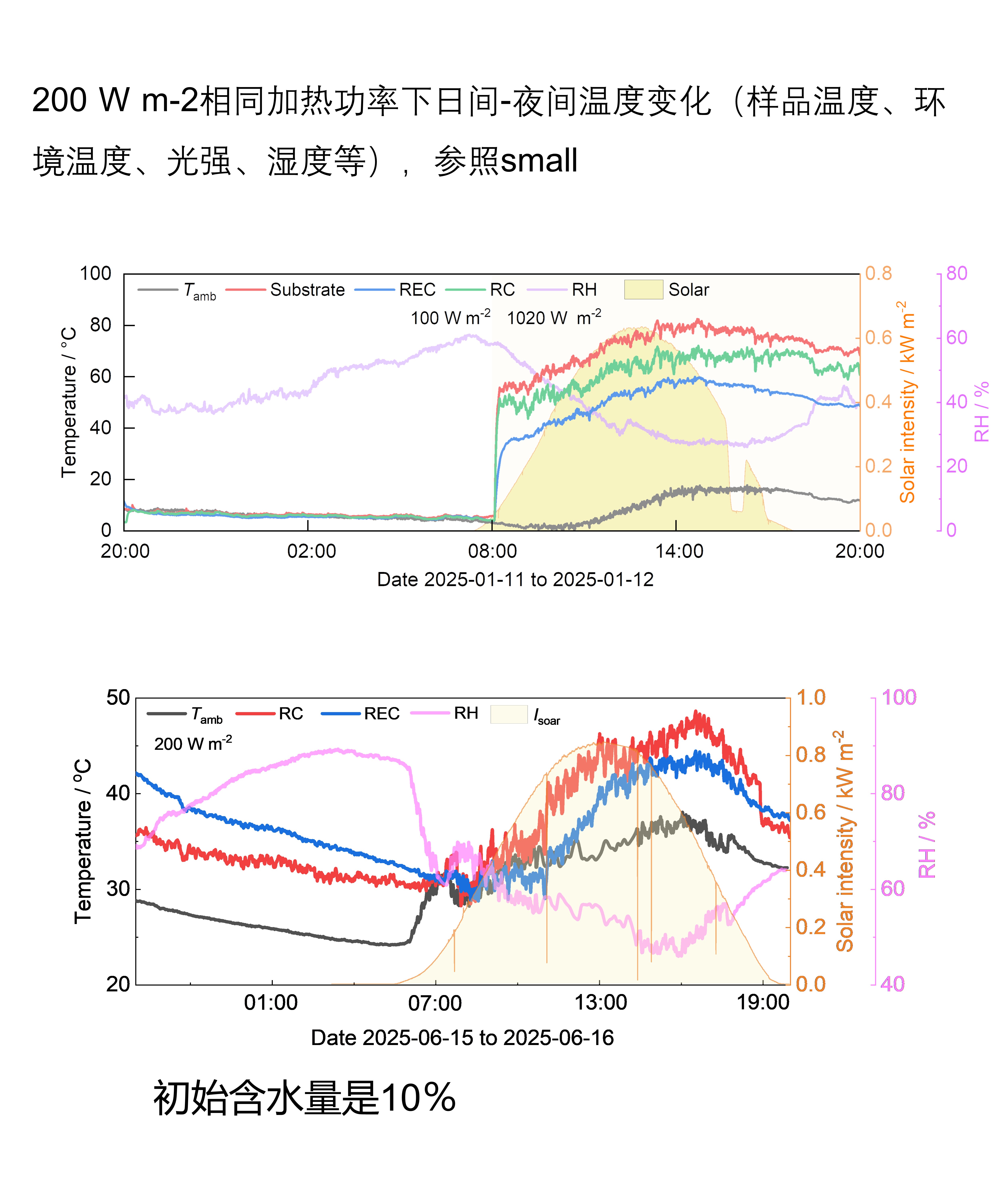


**Fig. S21** Continuous all-day cycle performance of REC hydrogel (initial water content 10 wt.%), and RC paint from 2025-06-15 to 2025-06-16 (*P*_heating_ = 200 W m^-2^ in the all day), including solar intensity, RH, and ambient temperature


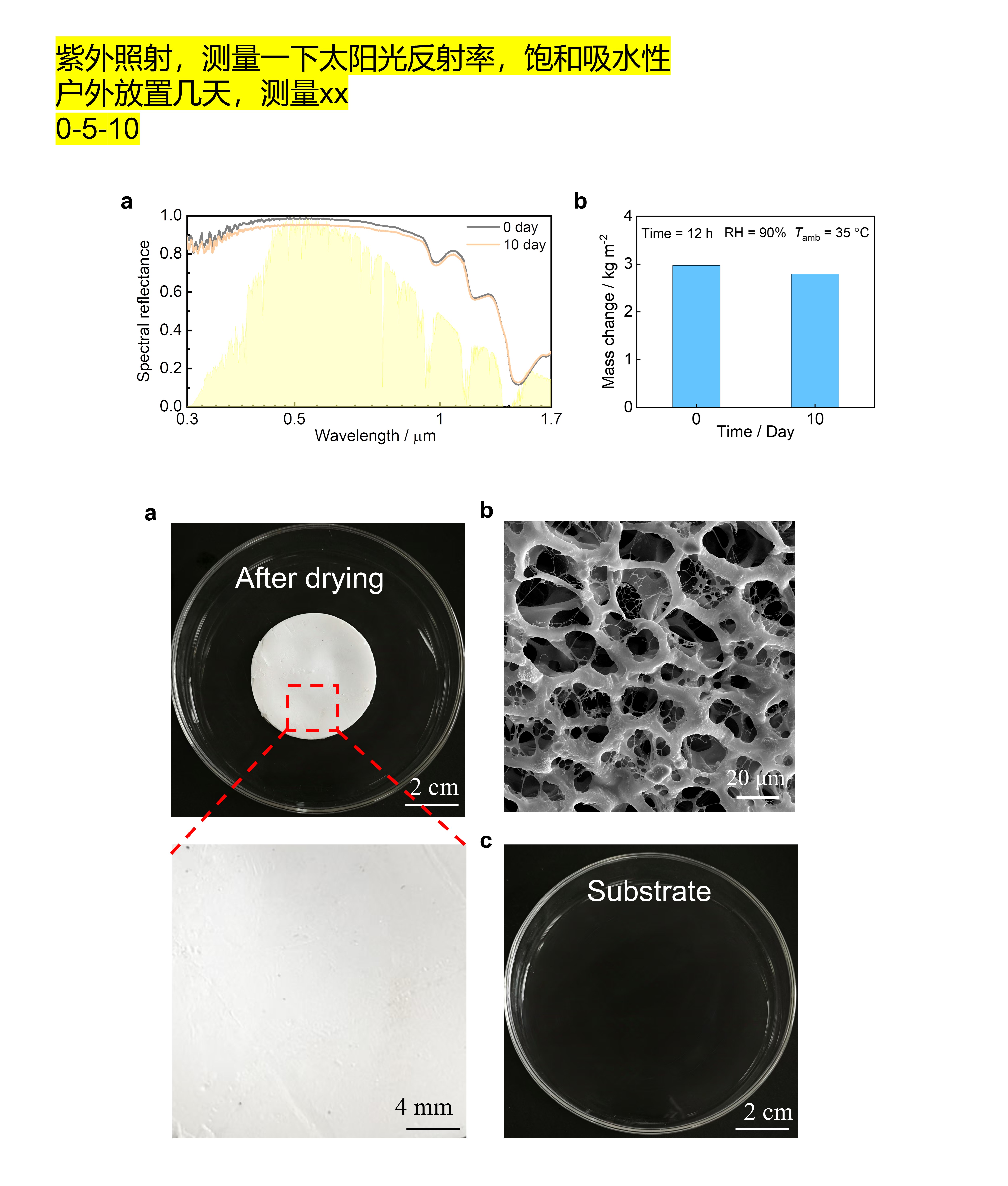


**Fig. S22 a** Optical images of the dried hydrogel, **b** its cross-section SEM image, and **c** the substrate


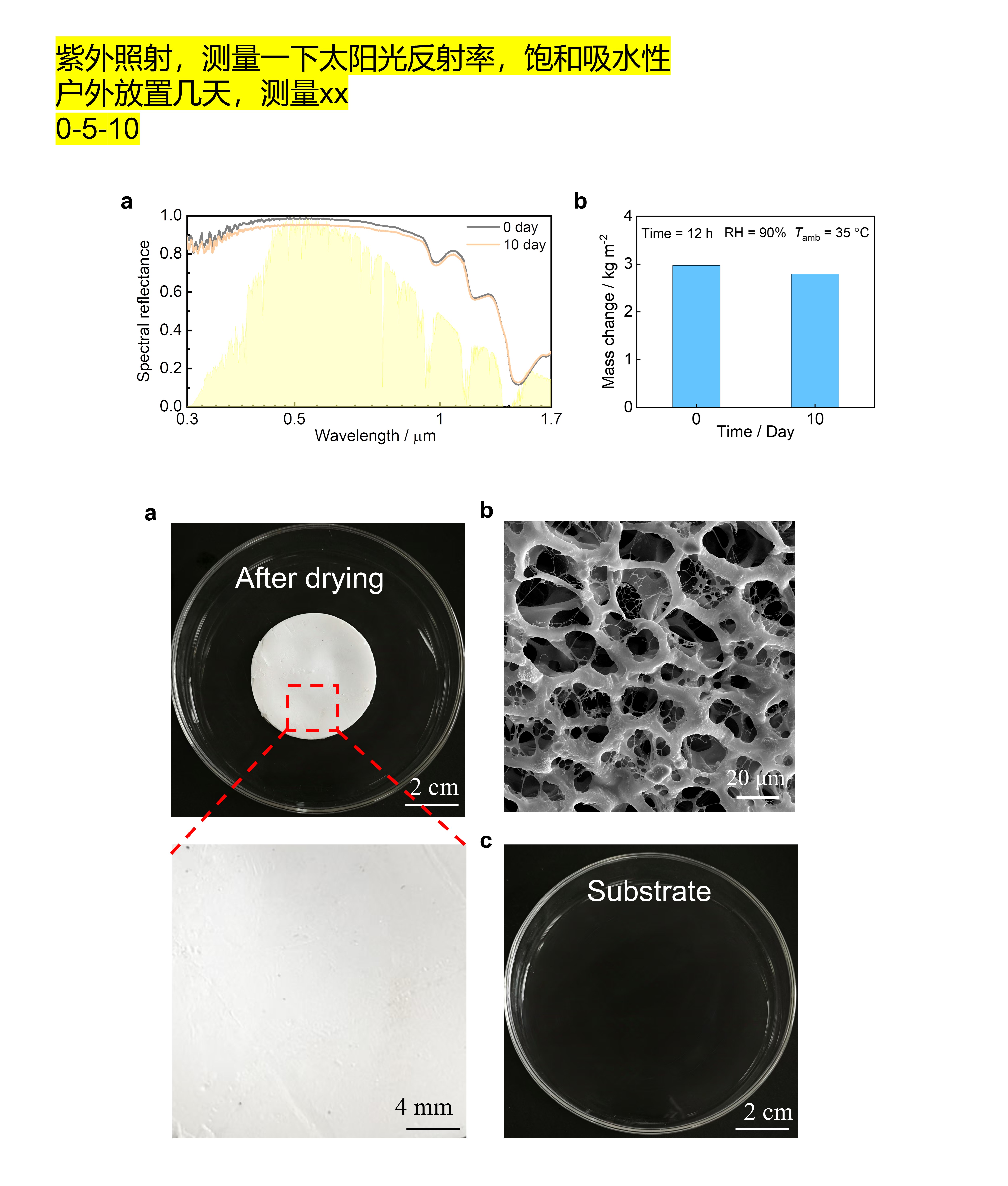


**Fig. S23** **a** Spectral reflectance and **b** water capture performance of photonic hydrogels after being placed outdoors for ten days


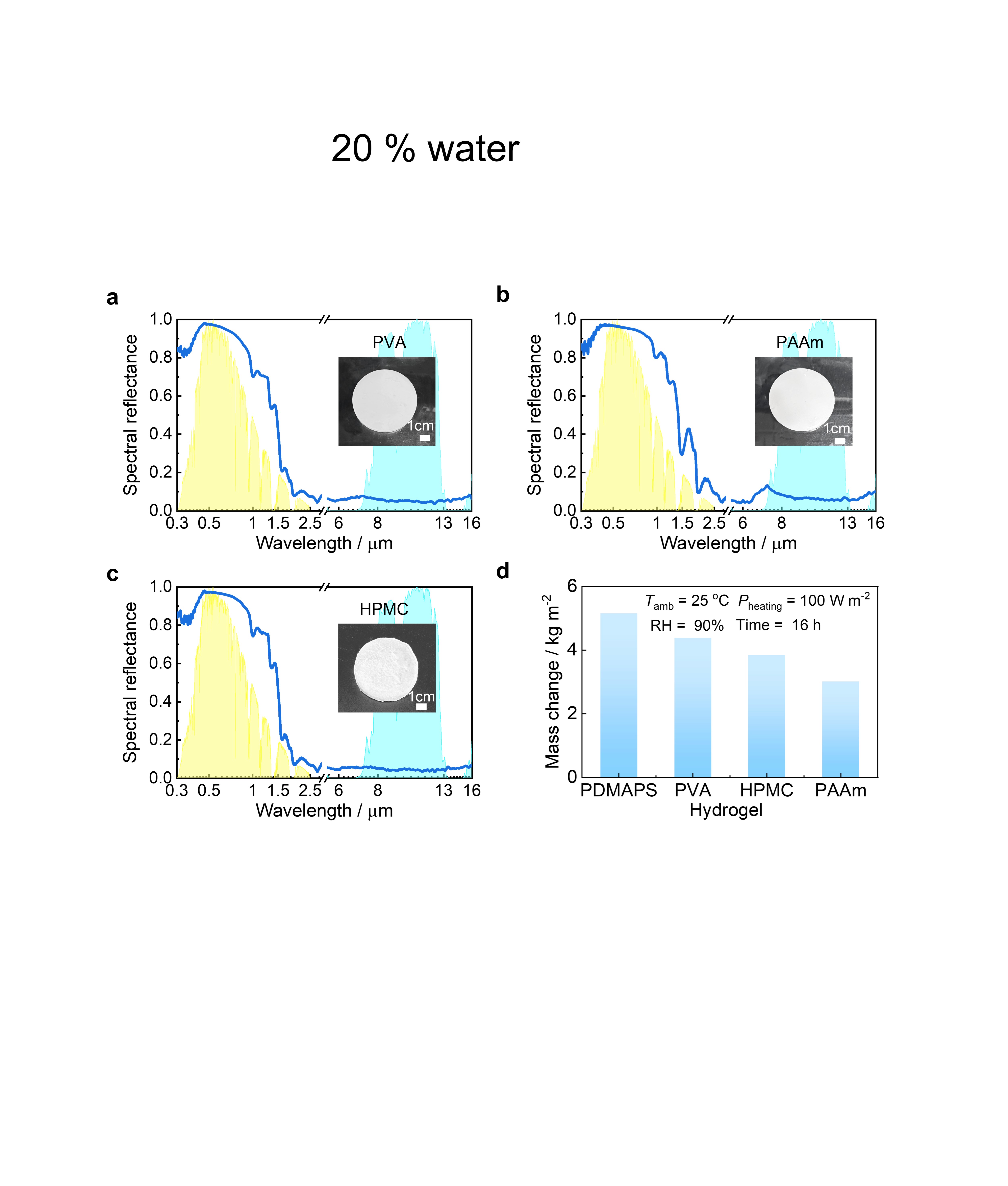


**Fig. S24** **a** to **c** Spectral reflectance and **b** water capture performance of different hydrogels at a water content of ~ 10 wt.%. Here, PVA, PAAm, and HPMC hydrogels were prepared based on ref. 1, 8, and 9

**Table S1** Average temperature in daytime and nighttime for the all-day experiment

|  | *T*_amb_ / ^o^C | *T*_substrate_ / ^o^C | *T*_REC_ / ^o^C | *T*_RC_ / ^o^C |
| --- | --- | --- | --- | --- |
| Daytime | 10.1 | 70.8 | 49.9 | 61.9 |
| Nighttime | 6.0 | 6.4 | 5.7 | 6.0 |

**Table S2** Total cost of materials in the design hydrogels

| Reagent | DMAPS | hBN | Al_2_O_3_ | APS | PEGDA | LiCl | Total |
| --- | --- | --- | --- | --- | --- | --- | --- |
| **Price /** $ m^-2^ mm^-1^ | 32.44 | 1.11 | 0.19 | 0.10 | 0.15 | 32.05 | 66.04 |

**Table S3** A comparison of cooling performance for different materials at sub-ambient and above-ambient conditions

| Refs. | Materials | Solar reflectance | Mid-infrared emittance | Temperature drop | Reference |
| --- | --- | --- | --- | --- | --- |
| [S10] | Bilayer  Aerogel-hydrogel | 0.98  (50 wt% h-BN) | 0.95 | 8.2 ^o^C | Ambient |
| [S11] | Bilayer  P(VdF-HFP)-P(VdF-HFP)@CaCl_2_ | 0.90 | 0.83 | 10.8 °C | Ambient |
| [S12] | Bilayer  Fiber-PVA@CaCl_2_ | 0.93 (Top)  0.78 (Bottom) | 0.94 | 10 °C | Ambient |
| [S13] | Single layer  ZrO2@PAAm-LiBr | 0.90  (without water) | 0.88 | 5.0 ^o^C | Ambient |
| [S14] | Singe layer  PDMS-S@LiCl | 0.92  (Visible) | 0.93 | 4.7°C | Ambient |
| [S15] | Single layer  Li-PAAm | None | None | 13−17 °C | Device substrate |
| [S16] | Single layer  PNAH | 0.98  (Transmittance) | 0.95 | 9.14 °C | Device substrate |
| This work | Single layer  NP@PDMAPS@LiCl | 0.87  (5% water) | 0.94  (5% water) | 20.9 ^o^C | Device  substrate |

**Supplementary References**

1. L. Yu, Y. Huang, Y. Zhao, Z. Rao, W. Li et al., Self-sustained and insulated radiative/evaporative cooler for daytime subambient passive cooling. ACS Appl. Mater. Interfaces **16**(5), 6513–6522 (2024). <https://doi.org/10.1021/acsami.3c19223>
2. C.H. Reitan, Surface dew point and water vapor aloft. J. Appl. Meteor. 1962 1982 **2**(6), 776–779 (1963). <https://doi.org/10.2307/26169602>
3. Algorithms and Technologies for Multispectral, Hyperspectral, and Ultraspectral Imagery XII. in *Society of Photo-Optical Instrumentation Engineers (SPIE) Conference Series* **6233**, (2006).
4. I. Ibrahim, D.H. Seo, A.M. McDonagh, H.K. Shon, L. Tijing, Semiconductor photothermal materials enabling efficient solar steam generation toward desalination and wastewater treatment. Desalination **500**, 114853 (2021). <https://doi.org/10.1016/j.desal.2020.114853>
5. Lienhard, J. H. I. & Lienhard, J. H. V. A Heat Transfer Textbook, fifth edition (2020).
6. G.W. Thomson, The antoine equation for vapor-pressure data. Chem. Rev. **38**(1), 1–39 (1946). <https://doi.org/10.1021/cr60119a001>
7. Q. Ye, N. Guo, M. Chen, Reversible solar heating and radiative cooling coupled with latent heat for self-adaptive thermoregulation. Appl. Phys. Lett. **126**(11), 113903 (2025). <https://doi.org/10.1063/5.0262028>
8. V. Penkavova, A. Spalova, J. Tomas, J. Tihon, Polyacrylamide hydrogels prepared by varying water content during polymerization: Material characterization, reswelling ability, and aging resistance. Polym. Eng. Sci. **62**(3), 901–916 (2022). <https://doi.org/10.1002/pen.25895>
9. K. Wang, S. Liu, J. Yu, P. Hong, W. Wang et al., Hofmeister effect-enhanced, nanoparticle-shielded, thermally stable hydrogels for anti-UV, fast-response, and all-day-modulated smart windows. Adv. Mater. **37**(14), 2418372 (2025). <https://doi.org/10.1002/adma.202418372>
10. X. Dong, K.-Y. Chan, X. Yin, Y. Zhang, X. Zhao et al., Anisotropic hygroscopic hydrogels with synergistic insulation-radiation-evaporation for high-power and self-sustained passive daytime cooling. Nanomicro Lett. **17**(1), 240 (2025). <https://doi.org/10.1007/s40820-025-01766-5>
11. Y. Sun, Y. Ji, M. Javed, X. Li, Z. Fan et al., Preparation of passive daytime cooling fabric with the synergistic effect of radiative cooling and evaporative cooling. Adv. Mater. Technol. **7**(3), 2100803 (2022). <https://doi.org/10.1002/admt.202100803>
12. J. Li, X. Wang, D. Liang, N. Xu, B. Zhu et al., A tandem radiative/evaporative cooler for weather-insensitive and high-performance daytime passive cooling. Sci. Adv. **8**(32), eabq0411 (2022). <https://doi.org/10.1126/sciadv.abq0411>
13. L. Xu, D.-W. Sun, Y. Tian, L. Sun, Z. Zhu, Self-rehydrating and highly entangled hydrogel for sustainable daytime passive cooling. Chem. Eng. J. **479**, 147795 (2024). <https://doi.org/10.1016/j.cej.2023.147795>
14. N. Xu, S. Ren, J. Chen, Q. Gou, G. Xue et al., Bio-inspired radiative-evaporative cooling polymer: a dual-functional composite for energy-efficient building cooling. Chem. Eng. J. **512**, 162301 (2025). <https://doi.org/10.1016/j.cej.2025.162301>
15. S. Pu, J. Fu, Y. Liao, L. Ge, Y. Zhou et al., Promoting energy efficiency *via* a self-adaptive evaporative cooling hydrogel. Adv. Mater. **32**(17), 1907307 (2020). <https://doi.org/10.1002/adma.201907307>
16. J. Shang, J. Zhang, Y. Zhang, X. Zhang, Q. An, Highly potent transparent passive cooling coating *via* microphase-separated hydrogel combining radiative and evaporative cooling. Nano Lett. **24**(23), 7055–7062 (2024). <https://doi.org/10.1021/acs.nanolett.4c01621>
